# FTO Inhibits Epithelial Ovarian Cancer Progression by Destabilising SNAI1 mRNA through IGF2BP2

**DOI:** 10.3390/cancers14215218

**Published:** 2022-10-25

**Authors:** Meige Sun, Xiaocui Zhang, Fangfang Bi, Dandan Wang, Xin Zhou, Xiao Li, Qing Yang

**Affiliations:** Department of Obstetrics and Gynecology, Shengjing Hospital of China Medical University, Shenyang 110004, China

**Keywords:** m6A, FTO, SNAI1, IGF2BP2, EMT, metastasis, ovarian cancer

## Abstract

**Simple Summary:**

Ovarian cancer is the gynaecologic malignant tumour with the highest mortality. Most patients are already in the advanced stage when they are found, and the 5-year survival rate is only 30–40%. Recurrence and metastasis of ovarian cancer are the difficulties of treatment. This study demonstrated for the first time that fat mass and obesity-associated protein (FTO) acts as an m6A demethylase to inhibit epithelial ovarian cancer growth and metastasis. Further, we demonstrated that FTO expression is negatively correlated with the FIGO stage in patients with epithelial ovarian cancer. In addition, we described the regulatory role of FTO-IGF2BP2-SNAI1 in epithelial ovarian cancer progression and provided a novel biomarker and treatment strategy for epithelial ovarian cancer.

**Abstract:**

Fat mass and obesity-associated protein (FTO) regulates critical pathways in various diseases, including malignant tumours. However, the functional link between FTO and its target genes in epithelial ovarian cancer (EOC) development remains to be elucidated. In this study, the biological functions of FTO were verified in vitro and in vivo. The m6A modification and the binding sites of SNAI1 mRNA were confirmed by m6A RNA immunoprecipitation (MeRIP) and RIP experiments. The actinomycin D assay was used to test the stability of RNA. We found that FTO was downregulated with increased m6A levels in EOC. Reduced expression of FTO was associated with a higher FIGO stage in patients with EOC. Mechanistically, FTO decreased the m6A level and stability of SNAI1 mRNA, causing downregulation of SNAI1 and inhibiting epithelial–mesenchymal transition (EMT). Furthermore, FTO-mediated downregulation of SNAI1 expression depended on IGF2BP2, which acted as an m6A reader binding to the 3′ UTR region of SNAI1 mRNA to promote its stability. In conclusion, FTO inhibits SNAI1 expression to attenuate the growth and metastasis of EOC cells in an m6A-IGF2BP2-dependent manner. Our findings suggest that the FTO-IGF2BP2-SNAI1 axis is a potential therapeutic target in EOC.

## 1. Introduction

Ovarian cancer is the deadliest gynaecological malignancy and ranks fifth among malignancies in women [1]. Epithelial ovarian cancer (EOC) is the most common pathological type. The global morbidity and mortality rates of ovarian cancer have continued to rise over the last 30 years [2]; 12,810 women have died from ovarian cancer in the United States alone in 2022 [1]. Patients with ovarian cancer have an insidious onset: early symptoms are not evident and are difficult to detect, and close to 80% of patients are at advanced stages when diagnosed [3]. Despite optimal surgery and chemotherapy, approximately 80% of patients with advanced ovarian cancer relapse after paclitaxel/platinum-based first-line chemotherapy, with a median progression-free survival of 12–18 months [3] and a five-year survival rate of 30–40% [4]. Therefore, it is essential to elucidate the critical mechanisms of ovarian tumourigenesis and progression to identify effective tumour diagnostic biomarkers and design new therapeutic approaches to improve prognosis.

N6-methyladenosine (m6A) is the most common internal mRNA modification in eukaryotes [5] and is enriched at sites with RRACH consensus sequences (R = G or A; H = A, C, or U) [6]. m6A modification is dynamically reversible, with methyltransferases (writers) catalysing m6A modification of adenosine on mRNA, demethylases (erasers) demethylating bases that have undergone m6A modification, and binding proteins (readers) that recognise the m6A-modified RNA and regulate target RNA degradation, localisation, and stability [7]. Many cancers exhibit alterations in the expression of m6A-related genes and cellular m6A levels. However, the oncogenic or tumour-suppressor roles of m6A are inconsistent across cancer types [8], and the role of m6A in EOC remains unclear.

Fat mass and obesity-associated protein (FTO) belongs to the non-heme Fe^2+^/α-ketoglutarate-dependent dioxygenase alkB family of proteins [9]. As the first m6A demethylase identified to be dysregulated in various tumours, FTO is upregulated in pancreatic, gastric, and bladder cancers and promotes tumour growth and metastasis [10,11,12,13,14,15,16,17,18,19,20,21]. Reduced FTO expression in the liver, prostate, papillary thyroid, colorectal, and lung adenocarcinoma has been associated with poor prognosis [22,23,24,25,26]. Mechanistically, FTO demethylates downstream target mRNAs, affects m6A levels, and exerts pro- or anti-cancer effects [27,28,29,30,31]. However, due to differences in FTO expression and reader selection, FTO has different impacts on tumorigenesis and progression. For example, FTO removes m6A modification of c-Myc mRNA to stimulate cell proliferation in leukaemia and cervical cancer cells [32,33]. Downregulated FTO in lung adenocarcinoma increases YTHDF1 binding to MYC mRNA, promotes MYC mRNA translation, and subsequently promotes tumour cell glycolysis, proliferation, and tumorigenesis [26]. In pancreatic cancer cells, inhibiting FTO expression reduces cell proliferation by increasing the m6A modification of the 3′ UTR region of platelet-derived growth factor-C (PDGFC) and regulates the degradation of PDGFC at the transcriptional level in an m6A-YTHDF2 dependent manner [10]. FTO can also promote pancreatic cancer progression by reducing the stability of TFPI-2 mRNA mediated by m6A-YTHDF1 [11]. FTO knockout suppresses the expression of c-Jun, JunB, and c/EBPβ, thereby impairing glycolysis and restoring CD8+ T cell function, which inhibits tumour growth [34]. However, the role of FTO in EOC progression and its underlying mechanisms are not clear. This study aimed to determine the anti-oncogenic role of FTO in regulating m6A RNA modification in EOC. 

## 2. Materials and Methods

### 2.1. Patient Samples

All clinical specimens were obtained from surgically resected tissues of patients between 2015 and 2017 at the Shengjing Hospital of China Medical University (CMU, Shenyang, China). None of the EOC patients had received radiation or chemotherapy before surgery. The material was collected after surgery. Paraffin-embedded sections of 5 normal ovarian tissues, 5 benign ovarian tumour tissues, 5 borderline ovarian tumour tissues, and 54 EOC tissues were used to test the expression of FTO by IHC. We randomly selected a certain number of samples from a total of 60 pairs of normal ovarian and EOC tissues to extract RNA and protein. The pathological diagnosis was confirmed by two clinical pathologists. The clinical stage of the patients was based on the International Federation of Gynecology and Obstetrics (FIGO) protocol. The Research Ethics Committee of the Shengjing Hospital of CMU approved the study, and all patients provided signed informed consent. The information on patient samples is listed in Table S3.

### 2.2. Bioinformatic Analyses

The mRNA expression profiles of FTO in normal ovarian tissue and EOC tissues were obtained using the HTseq-FPKM from the GTEx (https://xenabrowser.net/datapages/, accessed on 4 December 2020) and TCGA (https://portal.gdc.cancer.gov/, accessed on 4 December 2020) databases and further transformed by log2 (FPKM + 1) for gene expression level analysis. Proteomic data of FTO from the CPTAC database were visualised using the UALCAN website (http://ualcan.path.uab.edu/, accessed on 25 May 2021).

### 2.3. RNA Isolation and Reverse Transcription Quantitative Polymerase Chain Reaction (RT-qPCR)

Total RNA was extracted using TRIzol (Takara Bio, Kusatsu, Japan). The RNA samples were reverse-transcribed using HiScript II Q RT SuperMix for qPCR (+gDNA wiper) (Vazyme Biotech Co., Ltd., Nanjing, China). ChamQ Universal SYBR qPCR Master Mix (Vazyme) was used for RT-qPCR performed on an ABI 7500 Fast system. Primer sequences used in this study are listed in Table S1. GAPDH was used as an internal reference, and gene expression was analysed using the 2^−ΔΔCt^ method.

### 2.4. m6A Dot Blot Assay

After denaturation at 95 °C for 3 min, 500 ng of RNA per dot was loaded onto an Amersham Hybond-N+ membrane (Solarbio) and cross-linked at 37 °C for 30 min. The membrane was blocked with 5% non-fat milk for one hour. Then, the membrane was incubated with an m6A antibody (68055-1-Ig, Proteintech, Wuhan, China, 1:2000) overnight at 4 °C. The next day, the membrane was incubated with the secondary antibody for one hour at room temperature (RT) and detected using enhanced chemiluminescence (ECL) detection system (Thermo Scientific, Carlsbad, CA, USA). A 1% methylene blue solution was used as the loading control.

### 2.5. Western Blotting

Radioimmunoprecipitation assay (RIPA) lysis buffer (Beyotime, Shanghai, China) was used for protein extraction. Proteins were separated by 10% sodium dodecyl sulphate-polyacrylamide gel electrophoresis and transferred to a 0.22 μm PVDF membrane. The membrane was blocked with 5% non-fat milk and subsequently incubated with primary antibodies at 4 °C overnight. The primary antibodies used were as follows: GAPDH (10494-1-AP, Proteintech, Wuhan, China, 1:10,000), FTO (27226-1-AP, Proteintech, 1:3000), E-cadherin (20874-1-AP, Proteintech, 1:5000), N-cadherin (22018-1-AP; Proteintech, 1:3000), vimentin (60330-1-Ig, Proteintech, 1:10,000), ZEB1 (21544-1-AP, Proteintech, 1:3000), ZEB2 (14026-1-AP, Proteintech, 1:500), SNAIL (WL01863, Wanleibio, Shenyang, China, 1:500), SLUG (WL01508, Wanleibio, 1:500), and IGF2BP2 (11601-1-AP, Proteintech, 1:4000). The next day, the membrane was visualised using an ECL detection system after incubation with their respective secondary antibody. 

### 2.6. Immunohistochemistry (IHC)

Paraffin-embedded sections (4 μm thick) were used for IHC. After deparaffinisation, hydration, and antigen retrieval at RT, sections were incubated with antibodies specific for FTO (27226-1-AP, Proteintech, 1:500), SNAIL (WL01863, Wanleibio, Shenyang, China, 1:500), or Ki-67 (D2H10, Cell Signalling Technology, Boston, MA, USA, 1:800) at 4 °C overnight. On the second day, the sections were processed as described previously [35]. Two independent pathologists scored the specimens according to the percentage of positively stained cells (0 = 0–4%; 1 = 5–25%; 2 = 26–50%; 3 = 51–75%; 4 = 76–100%) and the intensity of staining (0 = none, 1 = slight, 2 = moderate, 3 = strong). The percentage and intensity were multiplied to obtain the total score of one visual field. The IHC score was equal to the average of the total scores for each visual field.

### 2.7. Cell Culture, Transfection, and Lentiviral Infection

All the cells were purchased from the Chinese Academy of Sciences Cell Bank (Shanghai, China) and were cultured in RPMI 1640 medium (Procell, Wuhan, China) supplemented with 10% foetal bovine serum (FBS; Procell) at 37 °C and 5% CO_2_ atmosphere. The A2780 and OVCAR3 cell lines were transfected with small interfering RNAs (siRNAs) targeting FTO, SNAI1, and IGF2BP1/2/3 from GenePharma (Suzhou, China). The siRNA sequences used are listed in Table S2. Lentiviruses overexpressing FTO or FTO shRNA (sh-FTO) were purchased from GeneChem (Shanghai, China). Plasmids that overexpress FTO wild-type (FTO-WT) and demethylation functional site mutant-type (FTO-MT) were purchased from GeneChem. Transfection was performed as previously described [35].

### 2.8. Cell Viability Assay 

After different treatments, 1.5 × 10^3^ cells were seeded in each well of 96-well plates. CCK-8 solution (10 μL; GK10001, GLPBIO) was added to each well and incubated for two hours. The absorbance was tested at 450 nm using a microplate reader every 24 h.

### 2.9. Cell Invasion Assay

Transwell chambers with 8-μm pores (Corning, New York, NY, USA) were used to perform Matrigel invasion assays. Medium (600 μL) containing 10% FBS was added to the lower chamber per 24 well. The upper chamber was precoated with Matrigel (BD Biosciences, San Jose, CA, USA) and loaded with 200 μL of serum-free medium containing 2 × 10^4^ cells. After 24 h of incubation, cells were fixed with 4% paraformaldehyde (PFA), stained with 5% crystal violet, photographed, and counted.

### 2.10. Wound Healing Assay

Cells were seeded and cultured in 6-well plates until 90% confluency, then a 200 μL sterile tip was used to draw a straight line in the middle of each well perpendicular to the bottom of the well plate and imaged with a microscope (Nikon, Japan). After incubation in FBS-free medium with 1 μg/mL Mitomycin C for 24 h, images were captured. 

### 2.11. EdU Assay

We used the BeyEOClick™EdU-555 cell proliferation kit (Beyotime Biotechnology, Shanghai, China) to investigate cell proliferation. The cells were cultured in a complete medium containing 10 µm EdU for two hours. The cells were fixed with 4% PFA and permeabilised with 0.3% Triton X-100, followed by incubation with a click reaction solution for 30 min in the dark. Images were captured using a fluorescence microscope (Nikon, Japan) and analysed using the ImageJ software (https://imagej.en.softonic.com, accessed on 1 December 2020).

### 2.12. m6A Methylated RNA Immunoprecipitation (MeRIP) 

According to the manufacturer’s protocol, we conducted the MeRIP assay using an EpiQuik CUT & RUN m6A RNA Enrichment (MeRIP) Kit (P-9018, Epigentek, Beijing, China). For simplicity, total RNA was incubated with m6A antibody or negative immunoglobulin G (lgG) at RT for 90 min, chemically cleaved, and subsequently captured by magnetic beads. RT-qPCR was performed as described previously. 

### 2.13. RNA Immunoprecipitation (RIP)

An RNA immunoprecipitation (RIP) kit (Bes5101, BersinBio, Guangzhou, China) and anti-IGF2BP2 antibody (11601-1-AP, Proteintech) were used to perform the RIP assay according to the manufacturer’s guidelines. Rabbit IgG was used as a negative control. RT-qPCR was conducted as previously described.

### 2.14. RNA Stability Assay

Once cultured cells reached 60–80% confluency, we added 10 µg/mL of actinomycin D (ACD, GC16866, GlpBio Technology, Shanghai, China) into the complete medium. Cellular RNA was extracted at 0, 3, and 6 h after incubation with ACD for RT-qPCR analysis. The half-life of the mRNA was calculated using the fitting curve formula.

### 2.15. In Vivo Growth Assays 

The ethics committee of Shengjing Hospital of CMU approved the animal research in our study (NO.2021PS410K). Female BALB/c nude mice were purchased from HFK Biosciences (Beijing, China). OVACR3 cells were subcutaneously injected into the right axilla of 5-week-old female BALB/c nude mice (1 × 10^6^ cells per mouse, n = 6 per group). Tumour volume was calculated as V = 1/2 × length × width^2^. After three-weeks of observation, the mice were euthanised. The xenograft tumours were fixed in 4% PFA for IHC or stored at −80 °C for RNA and protein extraction.

### 2.16. In Vivo Metastasis Assays 

OVACR3 cells (2 × 10^6^) were suspended in 200 μL of PBS and injected into the abdominal cavity of 4-week-old female BALB/c nude mice (n = 6 per group) from the right lower abdomen. We observed the state of the mice daily for 20 days after cell injection. If cachexia symptoms such as emaciation, loss of skin lustre, slow movement, difficulty in eating, or death occurred, the experiment was terminated immediately. The mice were euthanised and necropsied. The locations and sizes of the metastatic tumours were recorded.

### 2.17. Statistical Analysis

Statistical analyses were performed using GraphPad Prism 8.0 (La Jolla, CA, USA). Mean ± standard deviation (SD) represents the results of three independent experiments. Student’s *t*-test and chi-square tests were used to analyse the measurement and count data, respectively. Statistical significance was set at *p* < 0.05. 

## 3. Results

### 3.1. FTO Expression Decreased in EOC 

**Figure 1:**
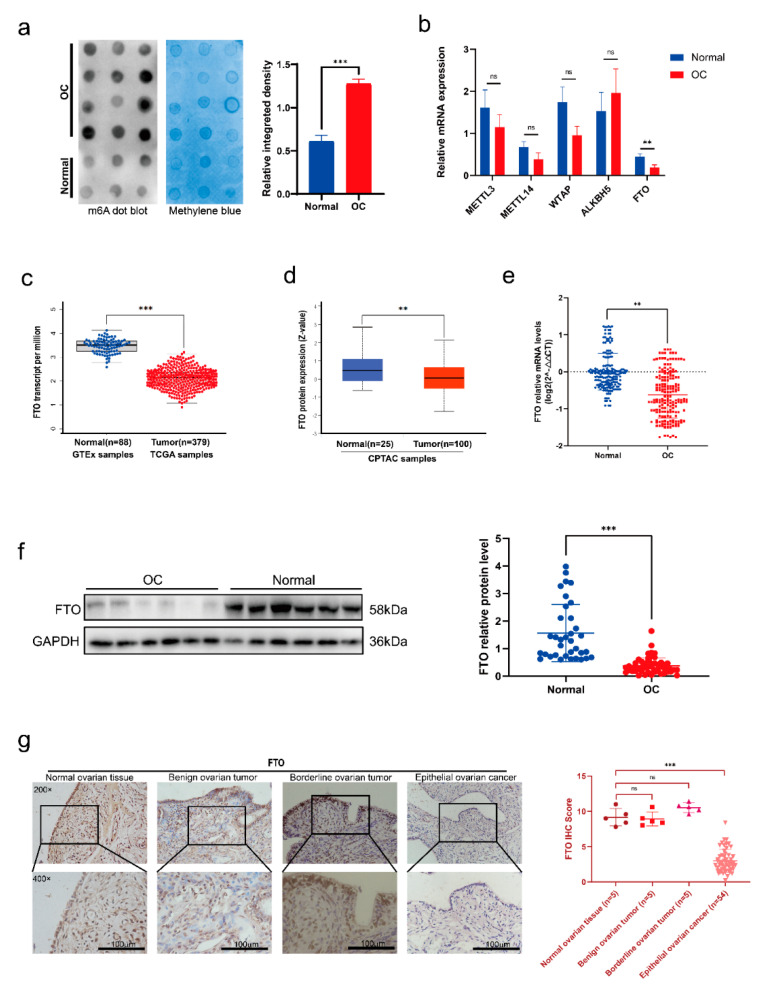
Lower expression of FTO in EOCs compared with normal ovarian tissues. (**a**) m6A levels of EOC and normal ovarian tissues by m6A dot blot. (**b**) Expression levels of the key enzymes of m6A in EOC and normal ovarian tissues by RT−qPCR. (**c**) The abundance of FTO transcripts from the TCGA database and the GTEX database. (**d**) Analysis of FTO protein level in the CPTAC database. (**e**) The mRNA level of FTO in 60 paired EOC and normal ovarian tissues detected by RT−qPCR. (**f**) FTO protein expression in 53 EOC and 35 normal ovarian tissues detected by Western blot. (**g**) FTO expression in normal ovarian tissues, benign, borderline epithelial tumours, and EOC detected by IHC assay. Scale bar, 100 μm. Data are presented as mean ± SD. ns, not significant; ** *p* < 0.01; *** *p* < 0.001. The uncropped blots are shown in Supplementary Materials File S1.

To clarify the role of m6A methylation in EOC, we first verified RNA m6A methylation levels in EOC and normal ovarian tissues. The m6A dot blot assay results showed increased m6A levels in the EOC tissues (Figure 1a). Next, we verified the gene level of m6A key enzymes in 30 pairs of EOC and normal ovarian tissues. Expression of the demethylase FTO was decreased significantly in EOC tissue compared to normal ovarian tissue, and there was no apparent difference in the expression of other enzymes (Figure 1b). We then carried out bioinformatic analyses for further verification of FTO expression in ovarian cancer. TCGA and GTEX database analyses showed that FTO mRNA expression was decreased in ovarian cancer patients (Figure 1c), and the CPTAC database showed that FTO protein levels were also decreased in ovarian cancer patients (Figure 1d). We expanded the sample size to 60 pairs, verified it with RT-qPCR, and found that the FTO mRNA was decreased in EOC tissues (Figure 1e). Western blot analysis further demonstrated downregulation of FTO protein in EOC tissue (Figure 1f). To compare the expression differences of FTO in various tissues, we used 5 normal ovarian tissues, 5 benign ovarian tumour tissues, 5 borderline ovarian tumour tissues, and 54 EOC tissues to test the expression of FTO by IHC. The FTO expression scores were similar in normal, benign, and borderline ovarian tumours, but were higher than those in EOC tissues (Figure 1g). Next, we analysed the expression levels in combination with the clinicopathological parameters (Table 1). All IHC sections of stages I–IV were derived from primary lesions. The primary lesion of patients in FIGO stages III–IV had lower FTO expression than those in stages I–II. We also compared the differences in FTO expression between serous EOC and other pathological types and found that the expression of FTO was not related to the pathological type. 

### 3.2. FTO Inhibits Malignant Phenotypes of EOC Cells In Vitro through Its Demethylation Function

**Figure 2:**
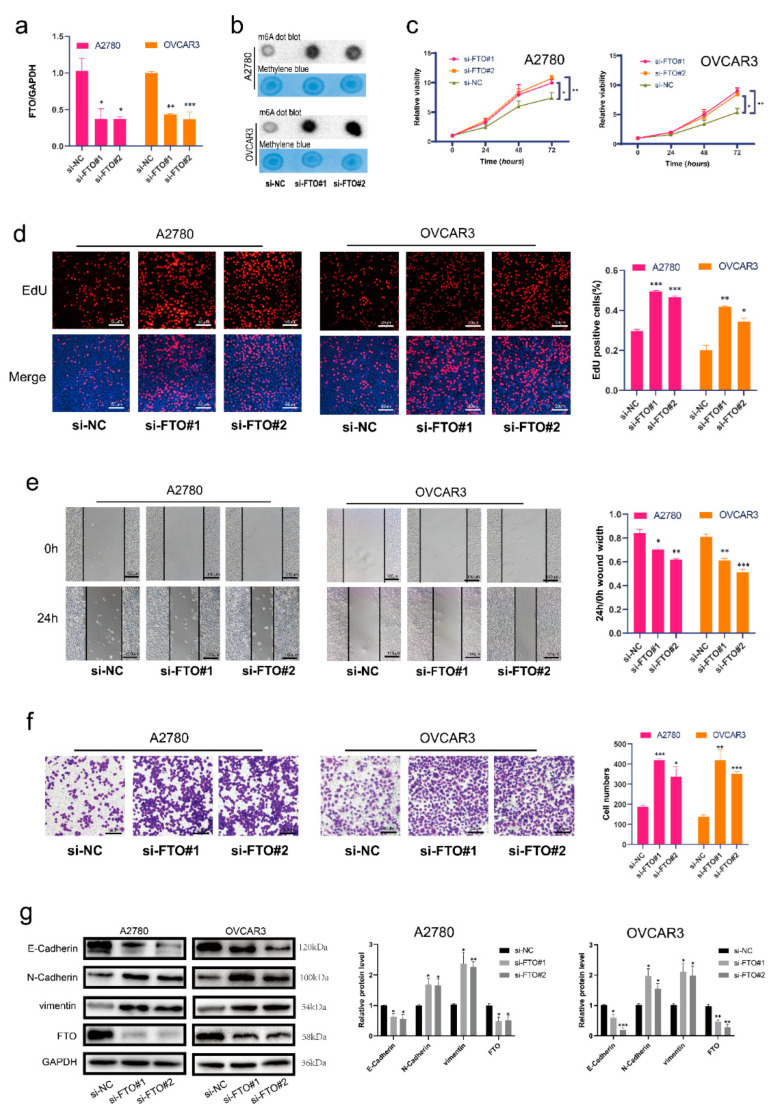
FTO knockdown promoted malignant phenotypes of EOC cells in vitro. (**a**) RT-qPCR validation of FTO knockdown in A2780 and OVCAR3 cells. (**b**) m6A levels of EOC cells were detected by m6A dot blot after FTO knockdown. (**c**) CCK-8 assays detected cell viability after FTO knockdown. (**d**) EdU assay detected the proliferation of EOC cells. Scale bar, 50 μm. (**e**) Wound healing assays detected cell migration after FTO knockdown. Scale bar, 100 μm. (**f**) Transwell assay tested the invasion of ovarian cancer cells. Scale bar, 50 μm. (**g**) Expression of EMT-related markers, N-cadherin, E-cadherin, and vimentin in FTO knockdown A2780 and OVCAR3 cells determined by Western blotting. The data represent the results of three independent replicates. Data are presented as mean ± SD; * *p* < 0.05; ** *p* < 0.01; *** *p* < 0.001. The uncropped blots are shown in Supplementary Materials File S1.

We first verified the FTO knockdown efficiency of siRNA in the A2780 and OVCAR3 cell lines (Figure 2a). Next, we used m6A dot blot assays to demonstrate that m6A levels were increased in the FTO knockdown group (Figure 2b). Cell proliferation and viability were also shown to increase after FTO knockdown (Figure 2c,d) via EdU and CCK-8 assays, respectively. The wound healing assay revealed that FTO knockdown upregulated the migration of EOC cells (Figure 2e). Transwell assays showed that FTO knockdown promoted EOC cell invasion (Figure 2f). In addition, we found decreased expression of E-cadherin and increased expression of N-cadherin and vimentin after FTO knockdown (Figure 2g).

**Figure 3:**
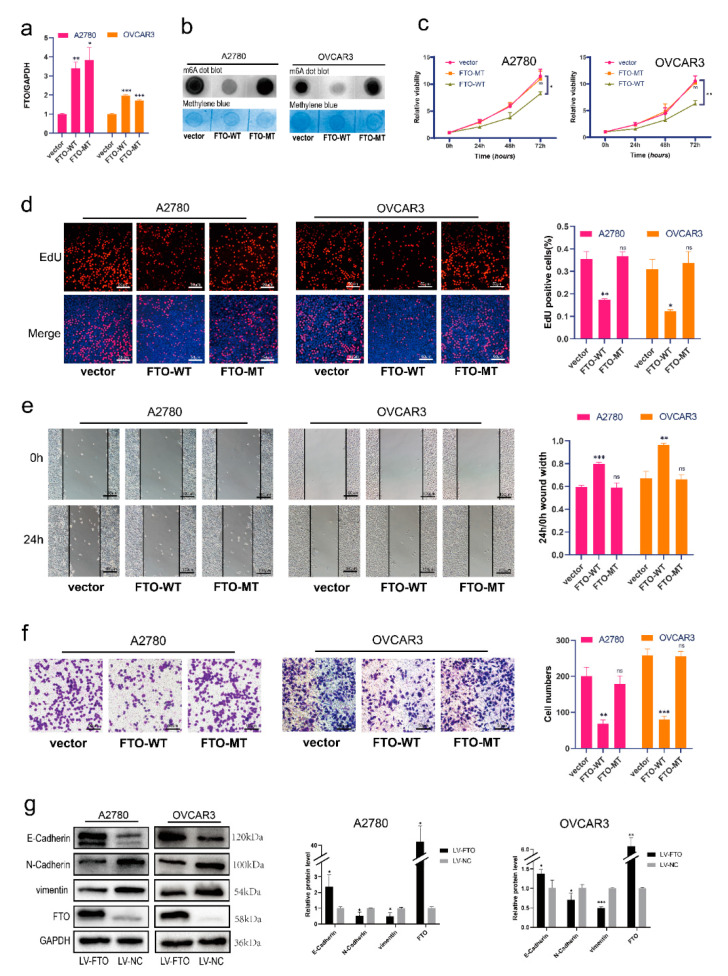
FTO overexpression inhibits malignant phenotypes of EOC cells in vitro. (**a**) RT-qPCR verified the efficiency of FTO-WT and FTO-MT overexpression in A2780 and OVCAR3 cells. After overexpression of FTO-WT and FTO-MT in A2780 and OVCAR3 cells, (**b**) m6A levels were detected by m6A dot blot; (**c**) CCK-8 assays tested cell viability; (**d**) EdU assay examined cell proliferation; scale bar, 50 μm; (**e**) The wound healing assay detected the migration; scale bar, 100 μm; (**f**) Cell invasion was evaluated by transwell assay; scale bar, 50 μm; (**g**) Expression of N-cadherin, E-cadherin, and vimentin in A2780 and OVCAR3 cells was determined by Western blot. The data represent the results of three independent replicates. Data are presented as mean ± SD; ns, not significant; * *p* < 0.05; ** *p* < 0.01; *** *p* < 0.001. The uncropped blots are shown in Supplementary Materials File S1.

Furthermore, we explored whether FTO suppresses ovarian cancer via its demethylation function. We transferred FTO wild-type (FTO-WT) and mutant-type (carrying H231A and D233A mutations, which disrupt the enzymatic activity of FTO; FTO-MT) [32] overexpression plasmids into A2780 and OVCAR3 cells (Figure 3a). The level of m6A decreased in FTO-WT-overexpressing EOC cells, but not in FTO-MT cells (Figure 3b). The viability of EOC cells decreased in the FTO-WT group, but not in the FTO-MT group (Figure 3c). EOC cell proliferation decreased after overexpression of FTO-WT but not FTO-MT (Figure 3d). Furthermore, EOC cells with FTO-WT overexpression had reduced cell migration capabilities; however, after overexpression of FTO-MT, the migration of EOC cells did not change (Figure 3e). Transwell experiments showed that overexpression of FTO-WT reduced the invasiveness of EOC cells; however, overexpression of FTO-MT did not affect the invasiveness of EOC cells (Figure 3f). We then infected ovarian cancer cells with an FTO overexpression lentivirus to construct stably transfected cell lines. After the overexpression of FTO, the expression of E-cadherin was increased, and N-cadherin and vimentin were downregulated (Figure 3g). Collectively, these results show that FTO attenuates the migration, invasion, proliferation, and EMT of EOC cells through its demethylation function.

### 3.3. FTO Inhibits the Expression of SNAI1 by Reducing the Stability of SNAI1 mRNA 

**Figure 4:**
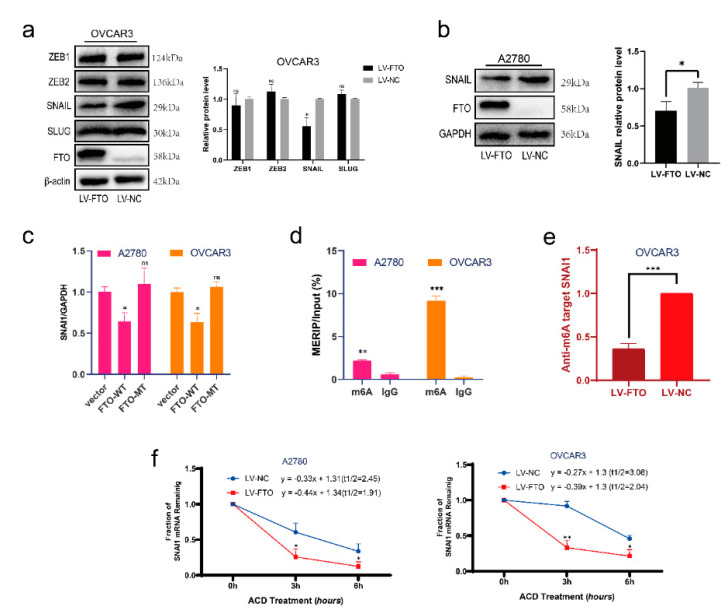
FTO decreases the stability and expression of SNAI1 mRNA. (**a**) Expression of EMT-related transcription factors, ZEB1, ZEB2, SNAIL and SLUG in OVCAR3 cells was determined by Western blot. (**b**) Expression of SNAIL in A2780 cells with indicated treatments detected by Western blot. (**c**) The expression of SNAI1 mRNA in FTO-WT overexpression, FTO-MT overexpression, and control vector transduced A2780 and OVCAR3 cells was examined by RT-qPCR. (**d**) MeRIP assay detected the m6A-modification on SNAI1 mRNA in A2780 and OVCAR3 cells. (**e**) The m6A level of SNAI1 mRNA in LV-FTO or LV-NC OVCAR3 cells was detected by MeRIP. (**f**) After treatment of cells with 10 μg/mL of ACD for 0, 3, and 6 h, SNAI1 mRNA level in LV-FTO or LV-NC cells was detected by RT-qPCR. Data are presented as mean ± SD; ns, not significant; * *p* < 0.05; ** *p* < 0.01; *** *p* < 0.001. The uncropped blots are shown in Supplementary Materials File S1.

To determine how FTO affects EMT, we verified the changes in several major EMT-related transcription factors after FTO in the OVCAR3 cell line. Western blotting results suggested that overexpression of FTO inhibited the expression of SNAIL (encoded by SNAI1), while the levels of ZEB1, ZEB2, and SLUG did not change (Figure 4a). Overexpression of FTO in the A2780 cell line also caused the downregulation of SNAIL (Figure 4b). To explore whether FTO acts on the transcriptional or translational level of SNAI1, we performed RT-qPCR experiments on cells transfected with FTO-WT, FTO-MT, and a control vector. SNAI1 expression in FTO-WT overexpressing cells decreased significantly, whereas SNAI1 expression in FTO-MT overexpressing cells did not change significantly (Figure 4c). We then performed an m6A RNA immunoprecipitation (MeRIP) assay on A2780 and OVCAR3 cell lines. We verified m6A modification on the mRNA of SNAI1 (Figure 4d). The m6A modification level of SNAI1 decreased after overexpression of FTO (Figure 4e). The actinomycin D experiment showed that the stability of SNAI1 mRNA decreased when FTO was overexpressed (Figure 4f).

### 3.4. FTO Reduces the Stability of SNAI1 mRNA with the Help of IGF2BP2

**Figure 5:**
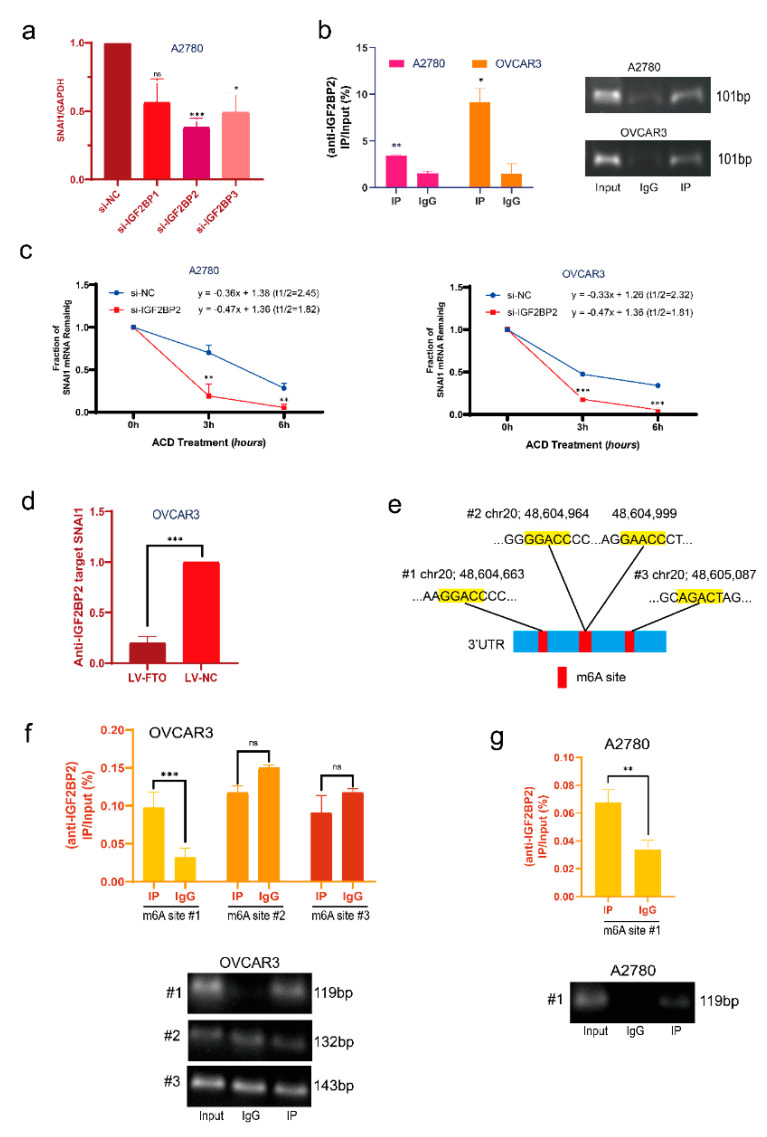
FTO decreases the stability and expression of SNAI1 mRNA via IGF2BP2. (**a**) SNAI1 mRNA expression in si−NC, si−IGF2BP1, si−IGF2BP2, and si−IGF2BP3 A2780 cells was examined by RT−qPCR. (**b**) Enrichment of SNAI1 mRNA interacting with IGF2BP2 was detected by RIP assay using IGF2BP2 specific antibody and IgG antibody, by RT−qPCR and DNA gel electrophoresis. (**c**) After treatment of cells with 10 μg/mL of ACD for 0, 3, and 6 h, SNAI1 expression in si−NC or si−IGF2BP2 cells was detected by RT−qPCR. (**d**) The level of SNAI1 mRNA interaction with IGF2BP2 detected by RIP assay using IGF2BP2-specific antibody in LV−FTO or LV−NC OVCAR3 cells. (**e**) m6A site of SNAI1 3′ UTR predicted by RMBase website. (**f**) Enrichment of SNAI1 m6A sites interacted with IGF2BP2 in OVCAR3 cells detected by RIP assay and by RT−qPCR. (**g**) Enrichment of SNAI1 m6A sites 1 interacted with IGF2BP2 in A2780 cells detected by RIP assay and by RT-qPCR. The data represent the results of three independent replicates. Data are presented as mean ± SD; ns, not significant; * *p* < 0.05; ** *p* < 0.01; *** *p* < 0.001. The uncropped blots are shown in Supplementary Materials File S1.

According to the literature, the IGF2BP protein family can bind m6A-modified mRNA and play an essential role in RNA stability [36]. Therefore, we used siRNA to knock down IGF2BP1/2/3 in the A2780 cell line and observed changes in SNAI1 mRNA expression. SNAI1 expression was more prominently decreased after IGF2BP2 knockdown (Figure 5a). Therefore, we believe that IGF2BP2 is the primary reader of the IGF2BP family that regulates the stability of SNAI1. Next, we used the RIP assay to verify that IGF2BP2 protein binds to SNAI1 RNA (Figure 5b). The actinomycin D experiment showed that the stability of SNAI1 mRNA decreased after IGF2BP2 knockdown (Figure 5c). After FTO overexpression, the level of SNAI1 mRNA bound by IGF2BP2 decreased (Figure 5d). Collectively these results show that FTO reduces m6A modification of SNAI1 mRNA through demethylation, thus inhibiting the binding of IGF2BP2, resulting in a reduction in the stability of SNAI1 mRNA and decreased SNAI1 expression.

To further verify the mechanism of interaction between IGF2BP2 and SNAI1 mRNA, we investigated m6A sites on human SNAI1 mRNA that may be combined with IGF2BP2 on the RMBase website (http://rna.sysu.edu.cn/rmbase/, accessed on 13 July 2021). All m6A sites are located in the SNAI1 mRNA 3’ UTR region (Figure 5e). The RIP assay showed that IGF2BP2 mainly binds to m6A site 1 (chr20; 48,604,663) in the EOC cell line OVCAR3 (Figure 5f). The RIP experiment in A2780 cells also proved that IGF2BP2 was bound to m6A site 1 (Figure 5g). 

### 3.5. FTO Inhibits Malignant Phenotypes of EOC Cells by Inhibiting SNAI1 via IGF2BP2

**Figure 6:**
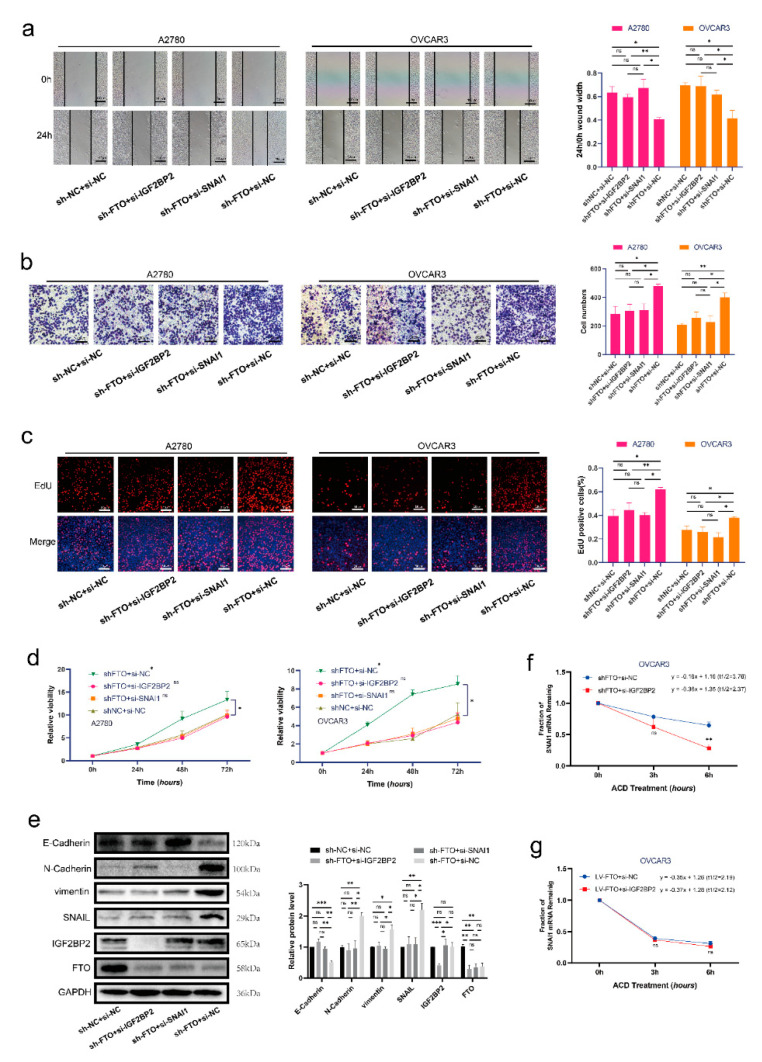
FTO exerts anti-tumour roles of EOC by inhibiting SNAI1 with the help of IGF2BP2. Wound healing (**a**), Transwell (**b**), EdU (**c**) and CCK-8 (**d**) assays were used to detect the migration, invasiveness, proliferation and viability of A2780 and OVCAR3 cells, respectively. (**e**) Protein levels of FTO, IGF2BP2, SNAI1, N−cadherin, E−cadherin and vimentin in OVCAR3 cells, detected by Western blotting. (**f**,**g**) SNAI1 mRNA stability in OVCAR3 cells was detected by ACD assay. The data represent the results of three independent replicates. Data are presented as mean ± SD. ns, not significant; * *p* < 0.05; ** *p* < 0.01; *** *p* < 0.001. The uncropped blots are shown in Supplementary Materials File S1.

To determine whether FTO plays an anti-tumour role by inhibiting the expression of SNAI1 in EOC, we first established A2780 and OVCAR3 cell lines stably transfected with anti-FTO shRNA (sh-FTO) lentivirus. Compared with the negative control group, the migration (Figure 6a), invasion (Figure 6b), proliferation (Figure 6c), and cell viability (Figure 6d) of sh-FTO cells were increased. Knockdown of IGF2BP2 in sh-FTO cells decreased their migration, invasion, proliferation, and viability. Knockdown of SNAI1 in sh-FTO cells also reversed the increased migration, invasion, proliferation, and cell viability. The actinomycin D experiment showed that the knockdown of IGF2BP2 in sh-FTO cells could reduce the mRNA stability of SNAI1 (Figure 6f). In contrast, knockdown of IGF2BP2 in FTO overexpressed cells did not change the mRNA stability of SNAI1 (Figure 6g). These results indicate that the function of IGF2BP2 in promoting mRNA stability is closely related to FTO expression. Furthermore, knockdown of IGF2BP2 reversed the increase in SNAI1 expression and EMT induced by FTO knockdown (Figure 6e).

### 3.6. FTO Inhibits the Growth and Metastasis of EOC In Vivo

**Figure 7:**
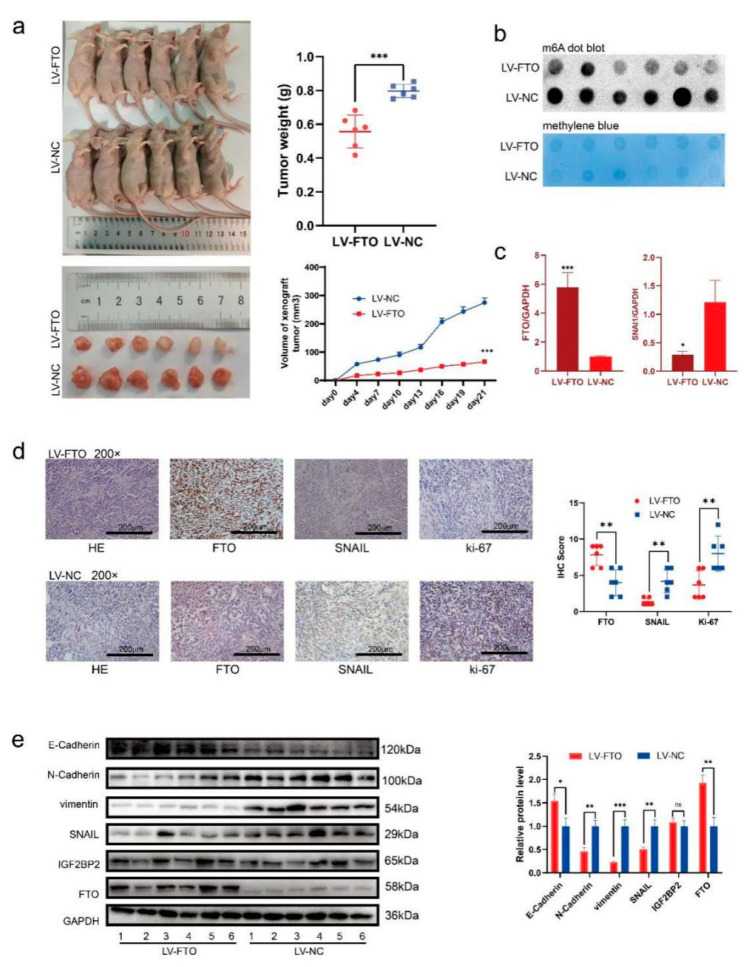
FTO inhibits EOC cell growth in vivo. (**a**) Representative pictures, the weight of xenograft tumours at the endpoints, and the tumour growth curves (n = 6 per group). (**b**) m6A level of xenograft tumours was detected by m6A dot blot. (**c**) RT-qPCR examined SNAI1 mRNA expression of xenograft tumours. (**d**) Representative images of IHC display the expression of FTO, SNAIL and Ki67 of xenograft tumours. Scale bar, 200 μm. (**e**) Western blot demonstrating the expression of EMT-related markers, FTO, IGF2BP2, and SNAIL. Data are presented as mean ± SD; * *p* < 0.05; ** *p* < 0.01; *** *p* < 0.001. The uncropped blots are shown in Supplementary Materials File S1.

To study the effect of FTO on the proliferation of EOC in vivo, we injected FTO stably overexpressed (LV-FTO) and negative control (LV-NC) OVCAR3 cell lines subcutaneously into nude mice. We observed slower tumour growth, decreased tumour volume, and reduced tumour weight in the LV-FTO group (Figure 7a). The m6A levels in subcutaneous xenograft tumours in the LV-FTO group decreased (Figure 7b), and the mRNA expression of SNAI1 was downregulated (Figure 7c). IHC showed decreased expression of SNAIL and Ki-67 in the LV-FTO group (Figure 7d). Furthermore, the expression of N-cadherin and vimentin decreased in the LV-FTO tumours. In contrast, E-cadherin expression was increased in the LV-FTO tumours (Figure 7e). The expression of IGF2BP2 did not change with FTO level (Figure 7e). Therefore, our results suggest that FTO can reduce the methylation level of m6A in EOC, inhibit SNAIL expression, and inhibit EMT in vivo.

**Figure 8:**
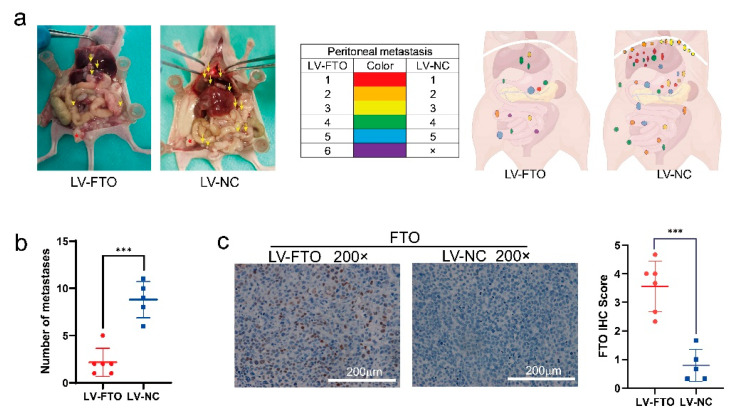
FTO inhibits EOC cell metastasis in vivo. (**a**) Tumour distribution at necropsy. Each colour represents the metastatic tumours from one mouse (n = 6). Red star indicates in situ lesion. Yellow arrow indicates metastatic focus. (**b**) The number at the endpoints of peritoneal xenograft metastatic tumours. (**c**) Representative images of IHC display the expression of FTO of peritoneal xenograft metastatic tumours. Scale bar, 200 μm. Data are presented as mean ± SD; *** *p* < 0.001.

Next, we constructed a model of abdominal metastasis of ovarian cancer. On the 25th day of modelling, one mouse in the LV-NC group died; therefore, we immediately terminated the experiment. After the euthanasia of surviving mice, we observed more intraperitoneal metastases in the LV-NC group than in the LV-FTO group, and the tumour distribution was more extensive (Figure 8a,b). The results of IHC proved that the expression of FTO in the LV-FTO group was significantly higher than that in the LV-NC group (Figure 8c). Together, these results indicated that FTO can weaken the growth and metastasis of EOC in vivo.

## 4. Discussion

This study demonstrated for the first time that FTO acts as an m6A demethylase to inhibit EOC growth and metastasis. Mechanistically, the decrease in FTO expression increased m6A modification in the 3′ UTR region of SNAI1 mRNA. An increase in m6A modification promoted the binding of IGF2BP2 to SNAI1 mRNA, thereby enhancing its stability and increasing SNAI1 protein expression, which promoted EMT. Our results elucidate the regulatory role of FTO-IGF2BP2-SNAI1 in EOC progression and provide a novel biomarker and treatment strategy for EOC.

Increasing evidence has shown that m6A is closely related to tumorigenesis and progression, but it may play contradicting roles [37]. On the one hand, the expression and activity of m6A enzymes affect m6A levels and their function in cancer. On the other hand, m6A regulates the expression of target genes, thereby promoting or inhibiting cancer progression. Previous studies have reported that m6A “writers” METTL3 and WTAP promote the occurrence and metastasis of EOC [38,39,40,41], while studies on the role of m6A “erasers” in EOC are inconsistent [42,43,44,45]. The present study demonstrated for the first time that decreased expression of the m6A demethylase FTO in EOC causes elevated m6A levels, and FTO expression is negatively correlated with the FIGO stage in patients with EOC. Mechanistically, FTO inhibits the expression of SNAI1 by acting as an m6A demethylase. Low FTO expression in EOC cells resulted in increased m6A levels of SNAI1 mRNA. The RNA-binding protein IGF2BP2 recognises and binds to the m6A site in the 3′ UTR region of SNAI1 mRNA, enhancing the stability of SNAI1 mRNA and increasing its mRNA and protein levels. Upregulated SNAI1 promoted EMT, proliferation, invasiveness, and migration of EOC cells. Therefore, we speculate that FTO may be a potential suppressor of EOC progression. However, the influence of the change of FTO expression on the m6A level of EOC, and whether FTO is independent of “writers” when it plays a suppressor role, needs to further expand the validation of clinical sample size and in-depth mechanism research. In addition, we also found that FTO decreased m6A levels in EOC cells and inhibited the growth and metastasis of EOC in vivo. These findings reveal fascinating complexities in epigenetic alterations and provide novel ideas and therapeutic options for EOC progression. The key factors that interfere with the expression of FTO and the possible drugs to restore the enzyme function of FTO need further exploration. Nevertheless, the role of FTO in peritoneal metastasis of EOC needs to be further verified by collecting clinical samples of peritoneal metastatic lesions. 

EMT is a significant driver of tumour malignancy and is strongly associated with tumour metastasis [46]. The role of m6A modifications in tumour development is manifested by the regulation of EMT [47]. METTL3 regulates β-catenin protein expression and membrane localisation in cervical, lung, and liver cancers, thereby promoting cell migration, invasion, and EMT [48]. Knockdown of METTL14 promotes ITGB4 expression via m6A-YTHDF2, stimulates EMT, and promotes migration, invasion, and metastasis in renal clear cell carcinoma cells [49]. In breast cancer cells, FTO deletion induces EMT by increasing m6A levels and altering the 3′-terminal processing of key mRNAs along the Wnt signalling cascade [50]. FTO also promotes EMT and breast cancer lung metastasis by regulating the translational extension of KRT7 mRNA via YTHDF1/eEF-1 [51]. The expression of SNAIL (Snail, SNAI1), a key transcription factor in EMT, has also been reported to be regulated by m6A in many diseases. In colorectal cancer, nasopharyngeal carcinoma, and hepatocellular carcinoma, METTL3 methylates Snail mRNA, thereby stabilising it and promoting tumour deterioration [52,53,54]. YTHDF1 mediates the translation of Snail mRNA with increased m6A in CDS, but not in the 3′ UTR, promoting cancer cell migration, invasion, and EMT in hepatocellular carcinoma [55]. Therefore, m6A may act as a promoter or inhibitor of EMT with tissue and functional specificity. In our study, low FTO expression increased the m6A modification of SNAI1 mRNA and promoted SNAI1 expression in ovarian cancer. As a critical transcription factor of EMT, the upregulation of SNAIL causes EMT enhancement in EOC cells. We constructed an in vivo peritoneal metastasis model to demonstrate the inhibitory effect of FTO on EOC metastasis. Our study revealed the regulatory effect and specific mechanism of FTO and m6A modifications on EMT in EOC and provided a new idea for developing intervention measures for EOC metastasis.

Moreover, the biological effects of m6A depend on different types of “readers”. YTHDF1 mainly improves the translation efficiency of target transcripts, YTHDC1 functions in pre-mRNA splicing and RNA export, and YTHDF2 mediates mRNA degradation [56]. In 2018, Huang et al. first identified the insulin-like growth factor 2 mRNA binding protein family (IGF2BPs) as a reading protein that recognises methylation-modified mRNAs distinct from YTH structural domain-containing proteins, mediating the enhanced stability of m6A-modified mRNAs [57]. IGF2BP2 was proved to act as an oncogene, and maintained FEN1 expression through an m6A- dependent mechanism in hepatocellular carcinoma cells [58]. As we have shown that alterations in m6A affect the stability of SNAI1 mRNA, we focused on the role of IGF2BPs in EOC development in an m6A-dependent manner. IGF2BPs are upregulated in most cancers and exert a pro-carcinogenic effect [59]. In this study, we found that IGF2BP2 binds to the m6A site of SNAI1 mRNA in EOC cells, resulting in enhanced mRNA stability of SNAI1. The increased SNAI1 expression and the enhanced proliferation, invasion, migration, and EMT of EOC cells caused by FTO knockdown could be eliminated by the knockdown of IGF2BP2. Together, we found that IGF2BP2 plays a vital role in m6A-mediated EOC promotion and that IGF2BP2 may be a potential therapeutic target for EOC. However, the effect of IGF2BP2 on EOC progression and the relationship between IGF2BP2 expression and EOC patient prognosis requires further exploration in the future. 

## 5. Conclusions

**Figure 9:**
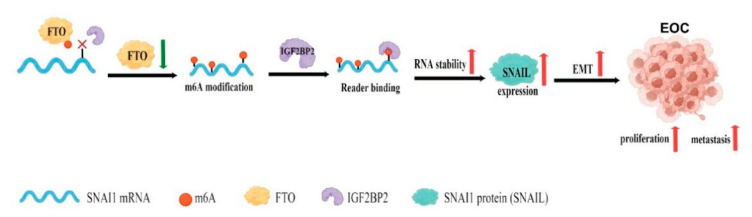
Schematic diagram of the present study (created with https://www.figdraw.com, accessed on 7 July 2022).

We demonstrated that FTO was expressed at low levels in EOC. The downregulation of FTO promoted EOC progression by accelerating the expression of SNAI1. Mechanistically, downregulation of FTO increases the m6A modification of SNAI1 mRNA and enhances the recognition and binding of IGF2BP2 to SNAI1 mRNA in an m6A-dependent manner, resulting in mRNA stabilisation and upregulation of SNAI1, promoting EMT, and the growth and metastasis of EOC cells (Figure 9). 

## Figures and Tables

**Table 1:** Analysis of FTO expression combined with clinicopathological parameters in EOC.

	Number (%)	FTO IHC Score (Average ± SD)	*p* Value
FIGO Stage			
I–II	17 (31.48%)	4.33 ± 1.66	***
III–IV	37 (68.52%)	2.37 ± 1.11	
Pathological type			
Serous	48 (88.89%)	3.03 ± 1.65	ns
Mucinous	1 (1.85%)	2.6	
Clear cell	2 (3.7%)	2.7	
Endometrioid	2 (3.7%)	3.6	
Poorly differentiated	1 (1.85%)	0.8	

ns: *p* ≥ 0.05, ***: *p* < 0.001.

## Data Availability

The data presented in this study are available in this article.

## Supplementary Materials

The following supporting information can be downloaded at: https://www.mdpi.com/article/10.3390/cancers14215218/s1, Table S1: Primer sequences; Table S2: transfection sequences; Table S3: The information of patient samples; Supplementary file S1: Raw pictures of blot.
