# Supplementary material for: FTO Inhibits Epithelial Ovarian Cancer Progression by Destabilising SNAI1 mRNA through IGF2BP2
Source: Cancers (Basel). 2022 Oct 25;14(21):5218. doi: 10.3390/cancers14215218 (PMC9658695; doi:10.3390/cancers14215218)

OVCAR3 (Left: LV-FTO, right: LV-nc)

vimentin

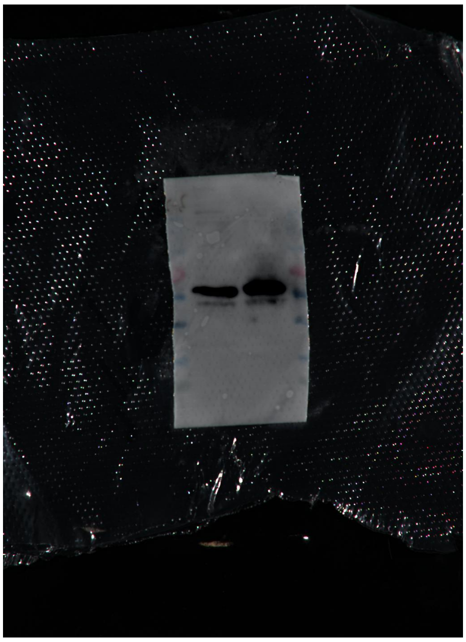

N-cadherin

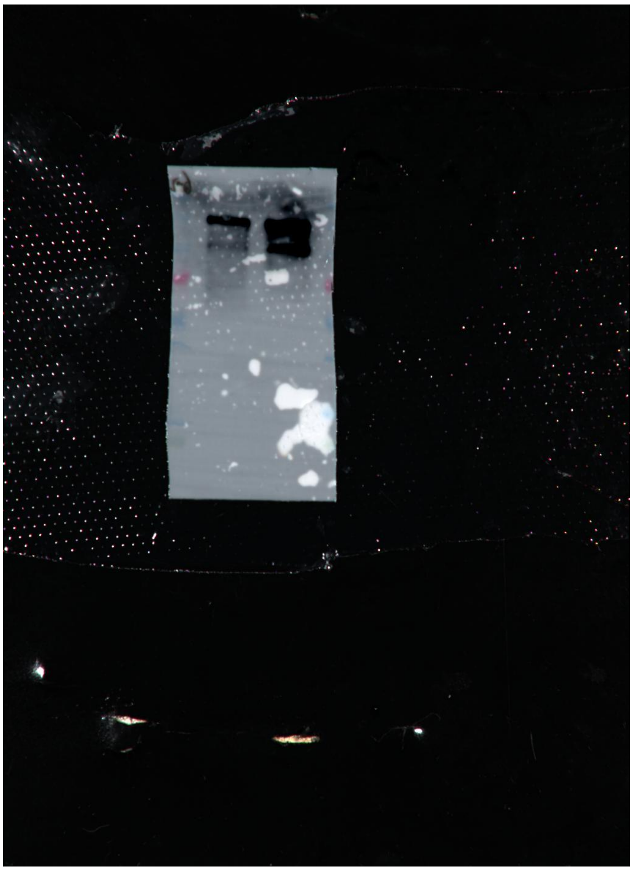

FTO(up)+GAPDH(middle)+SNAIL(down)

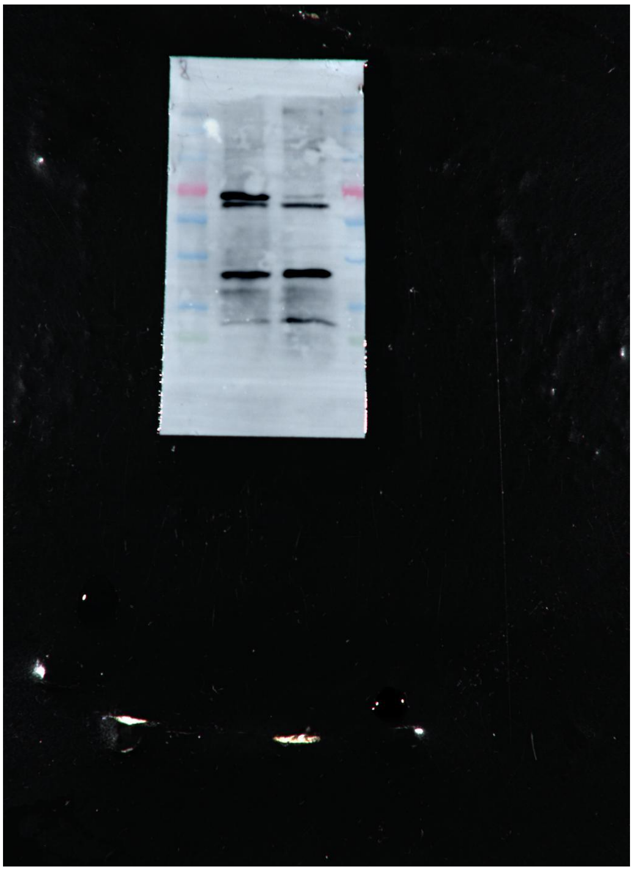

E-cadherin(up)+GAPDH(down)

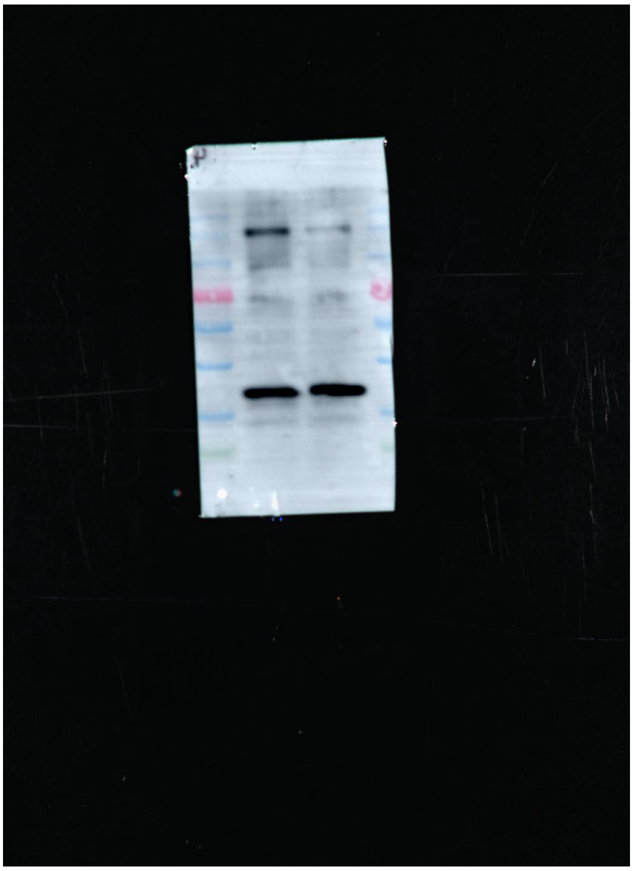

A2780 (Left: LV-FTO, right: LV-nc)

vimentin

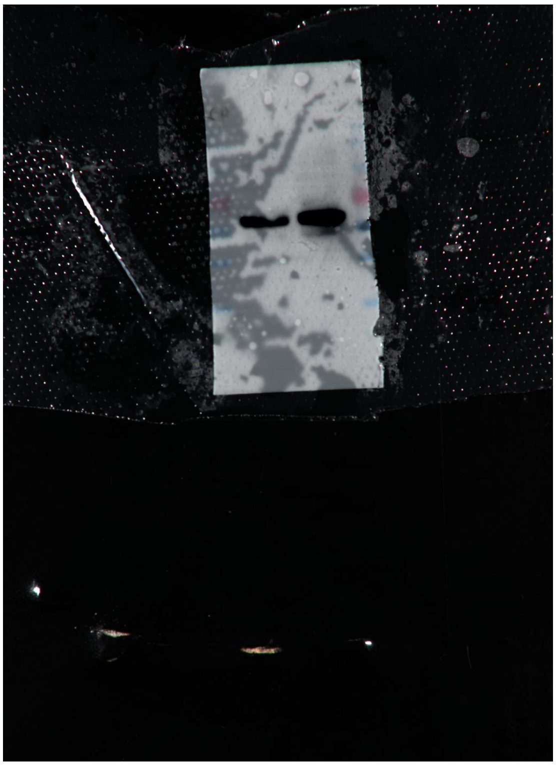

N-cadherin

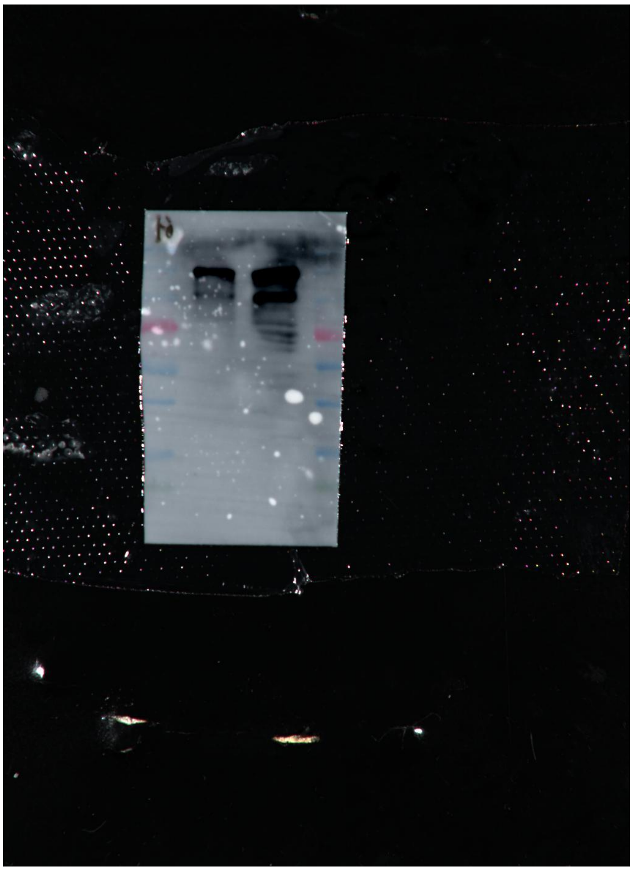

FTO(UP)+SNAIL(down)

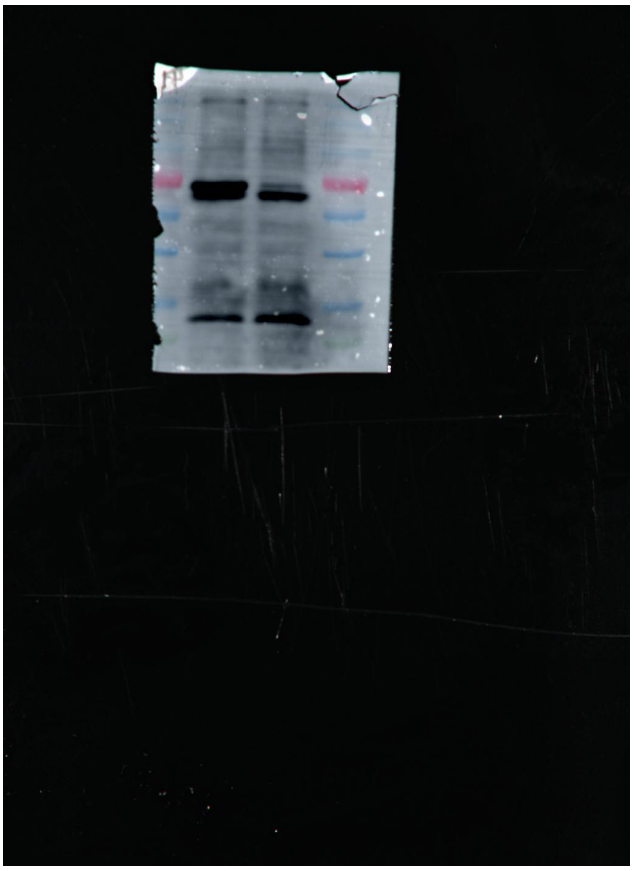

E-cadherin(up)+GAPDH(down)

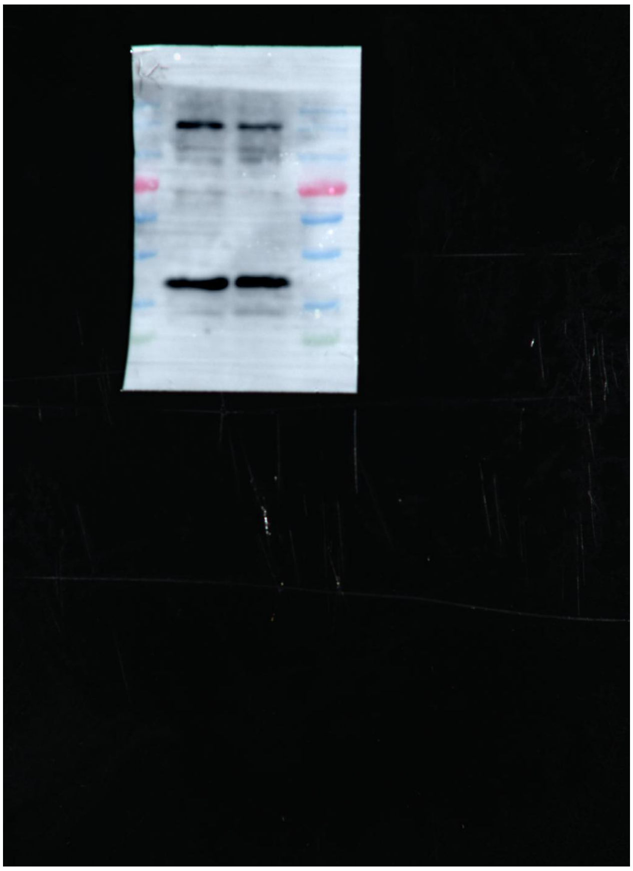

OVCAR3 (From left to right: si-NC, si-FTO#1, si-FTO#2)

N-cadherin

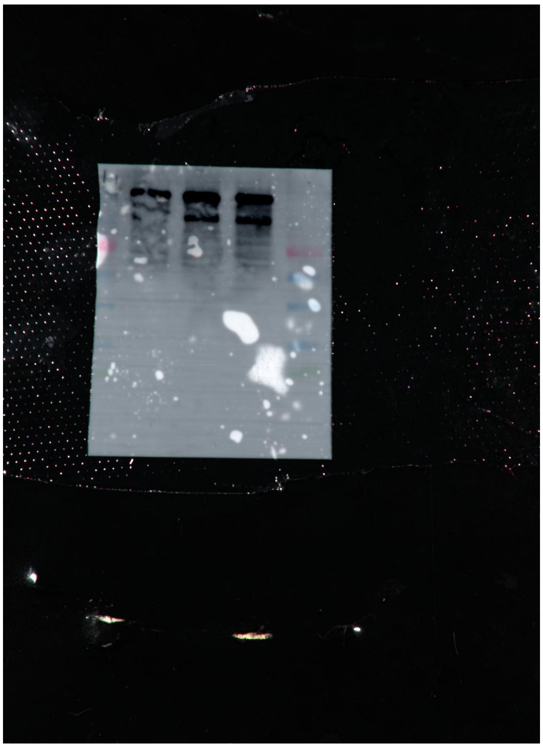

GAPDH

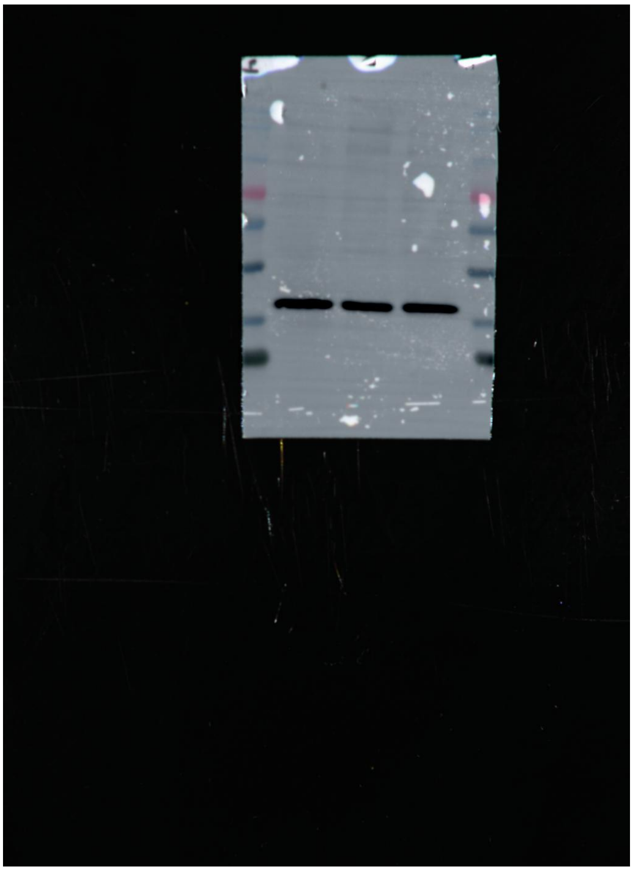

FTO

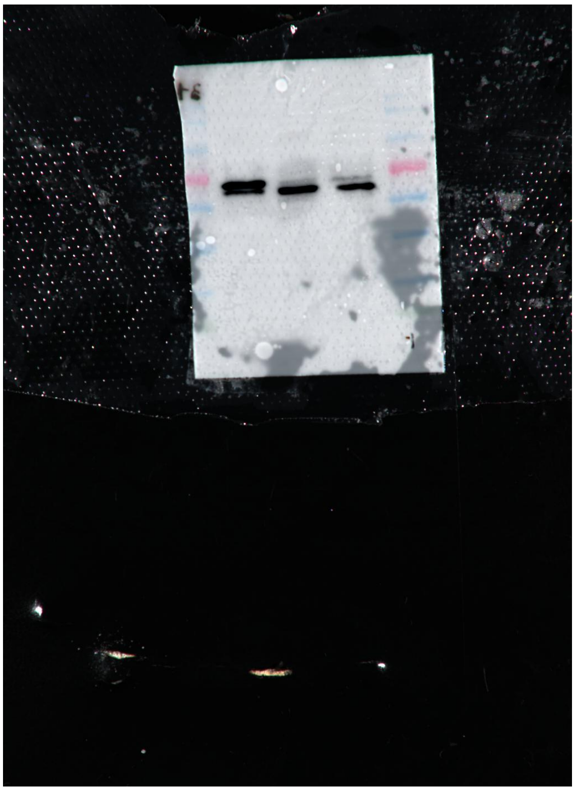

E-cadherin(up)

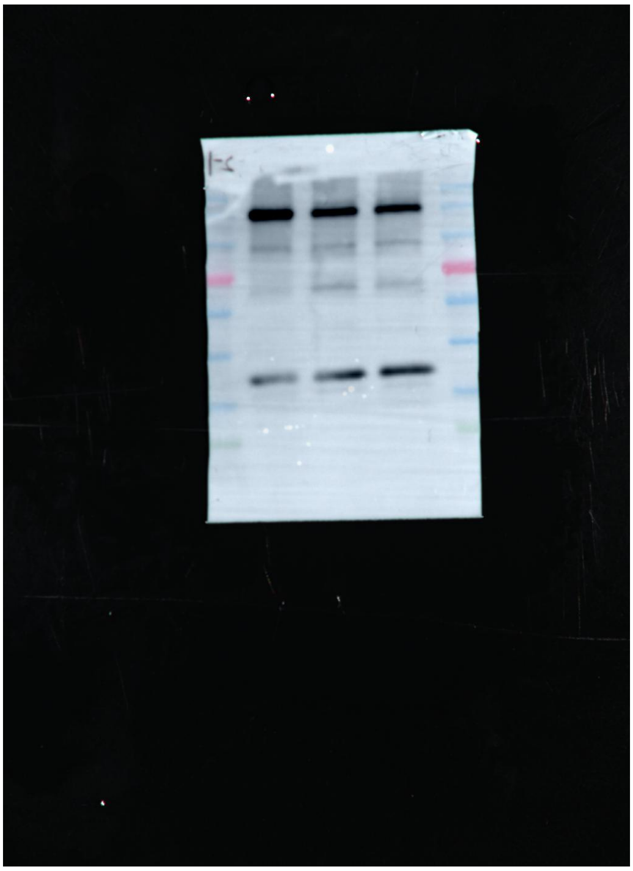

vimentin

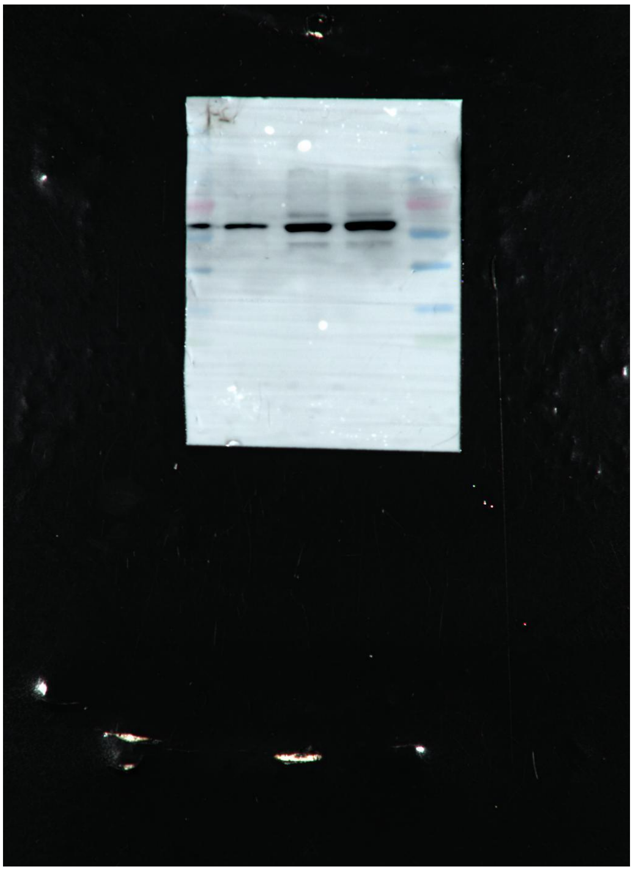

A2780(From left to right: si-NC, si-FTO#1, si-FTO#2)

vimentin

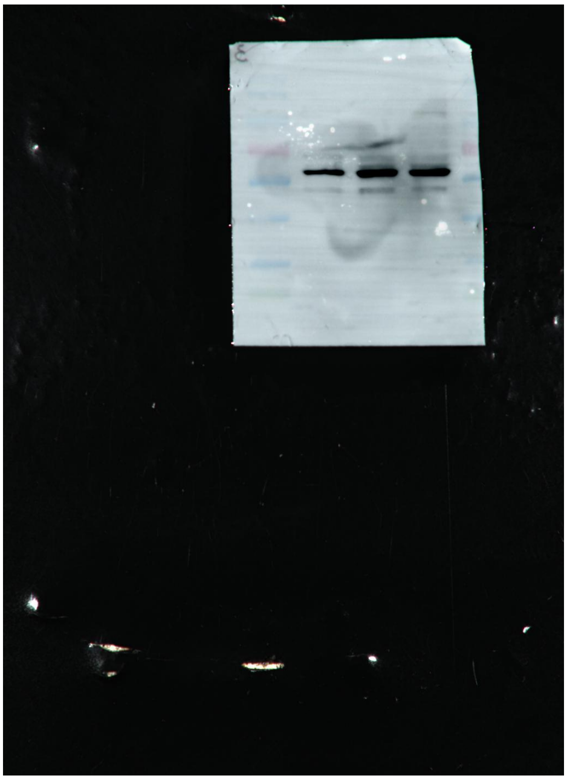

N-cadherin(up)

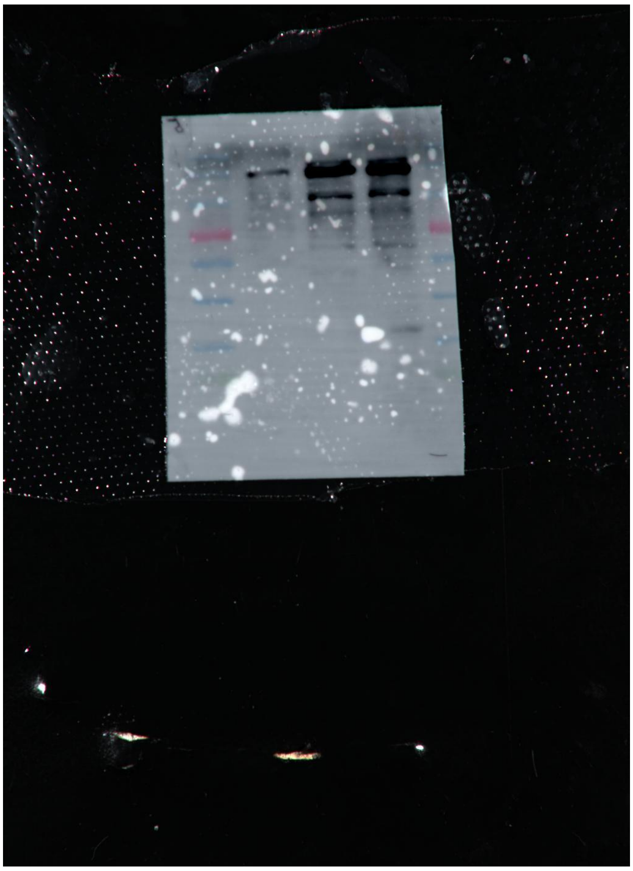

GAPDH

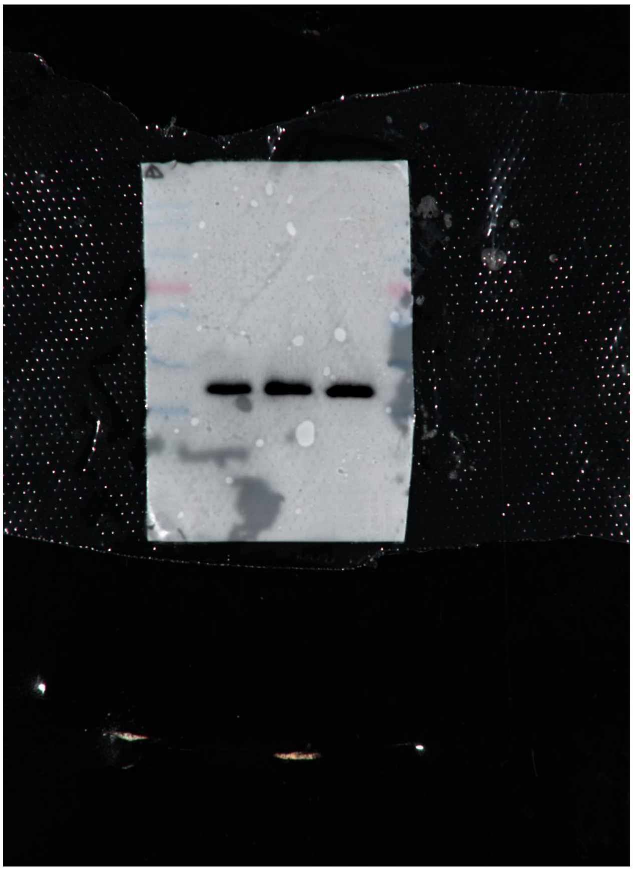

FTO

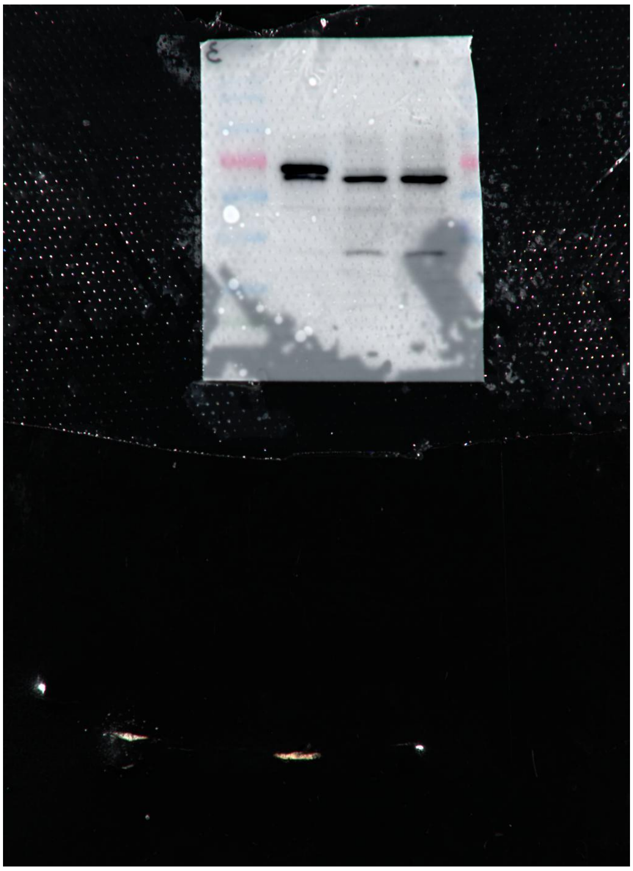

E-cadherin(up)

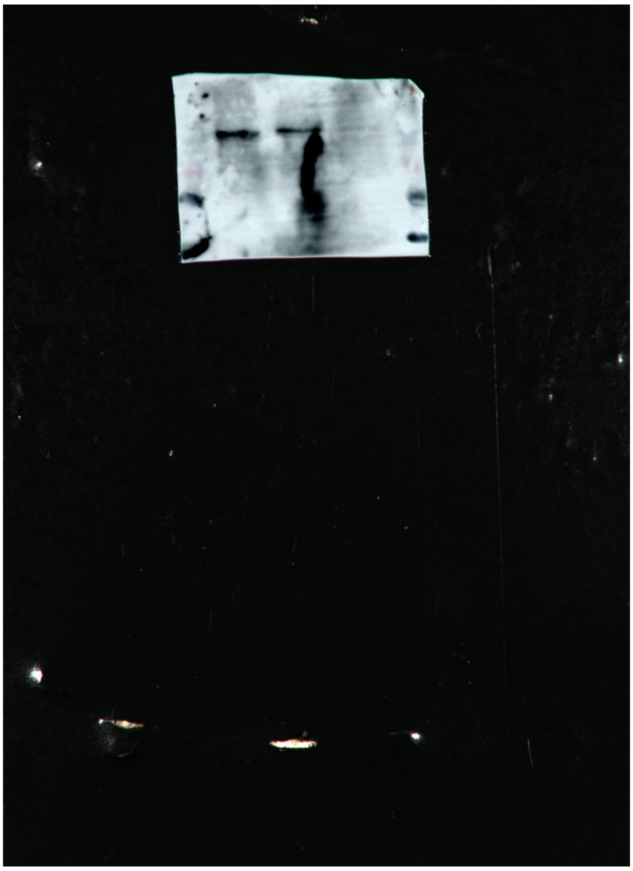

Figure1a

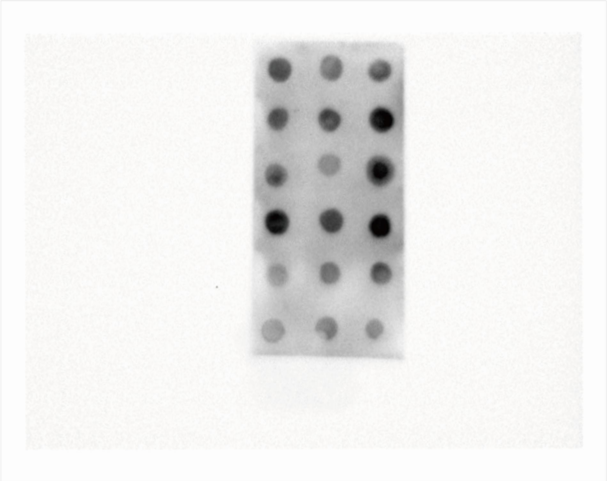

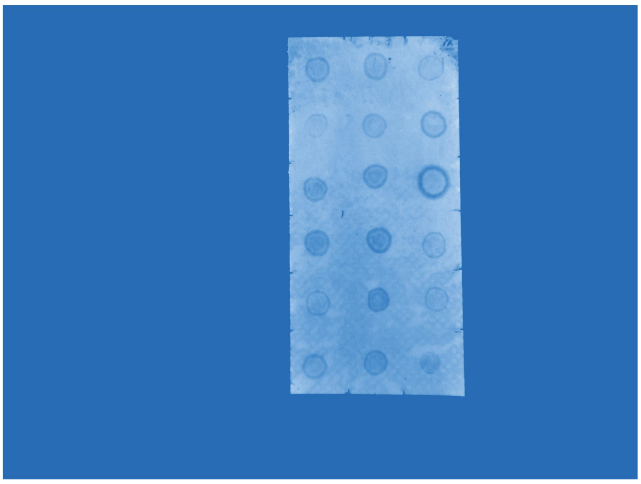

Figure1f

FTO

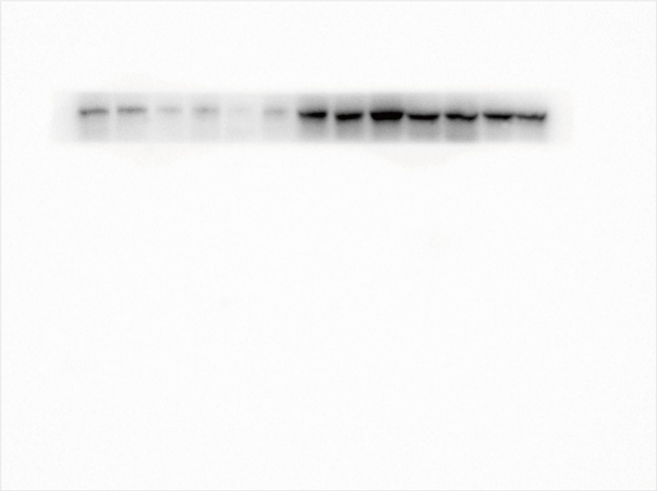

GAPDH

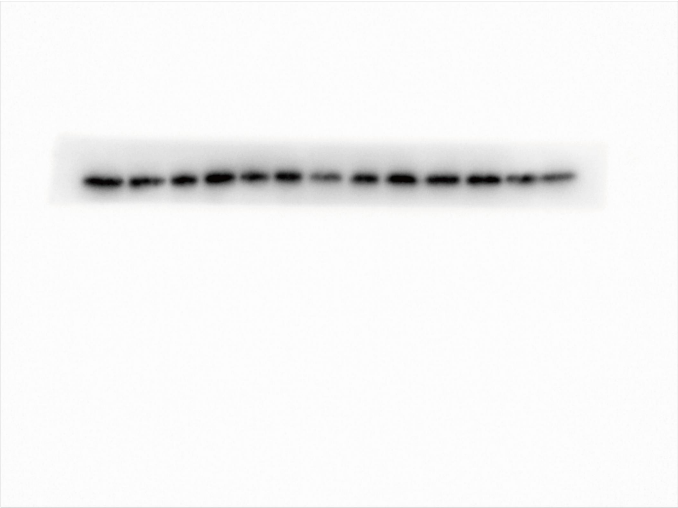

Figure 2b

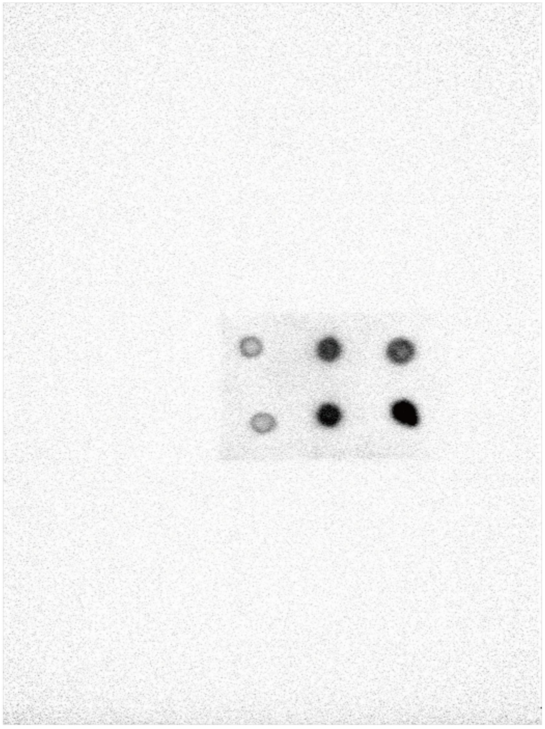

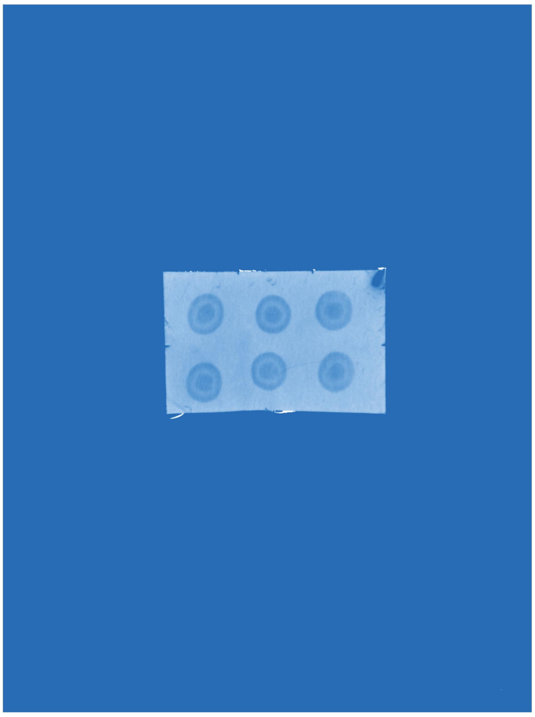

Figure 2g

A2780

E-cadherin

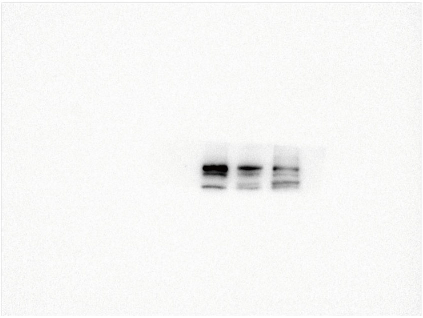

N-cadherin

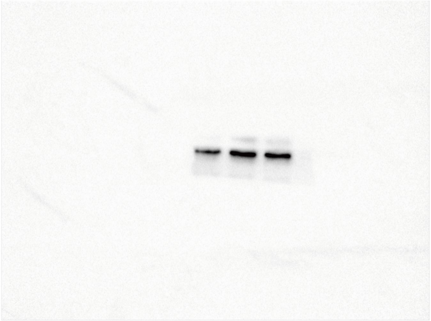

vimentin

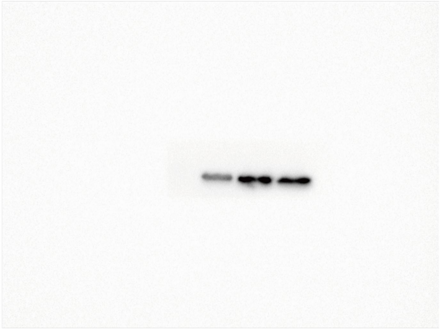

FTO

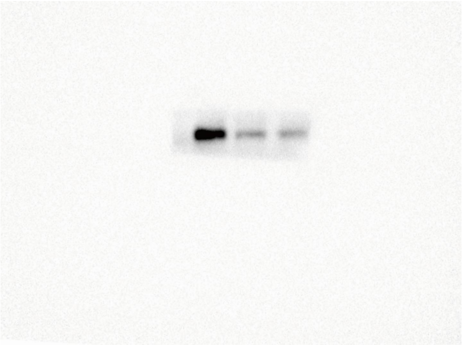

GAPDH

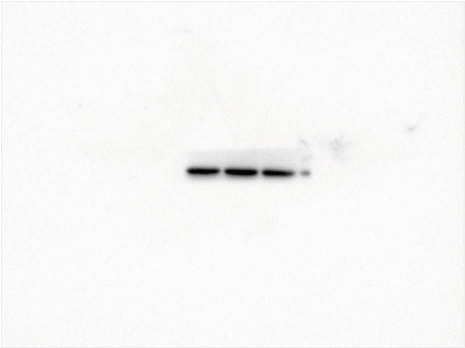

Figure 2g
OVCAR3
E-Cadherin

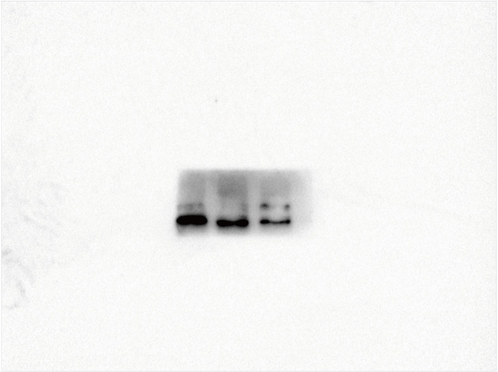

N-cadherin

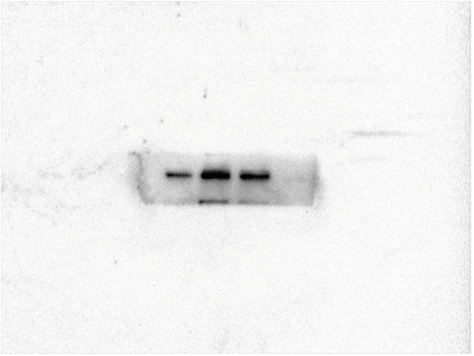

vimentin

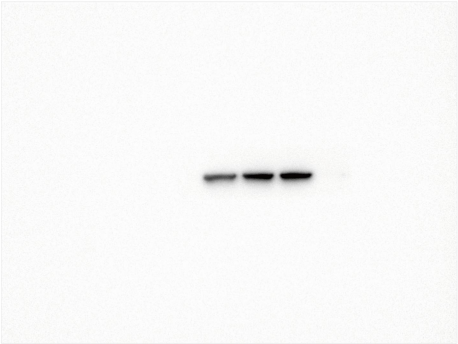

FTO

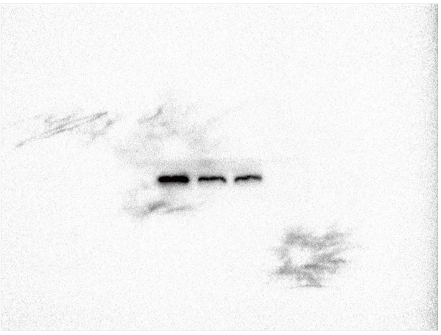

GAPDH

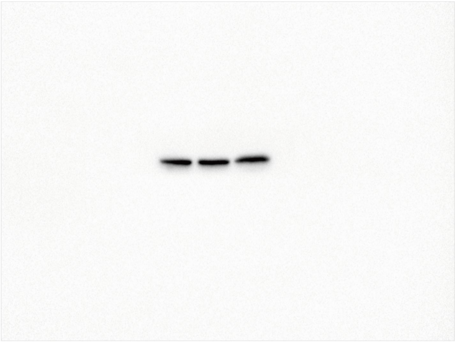

Figure 3b

A2780

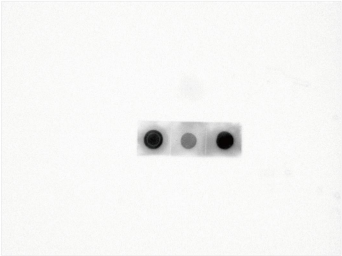

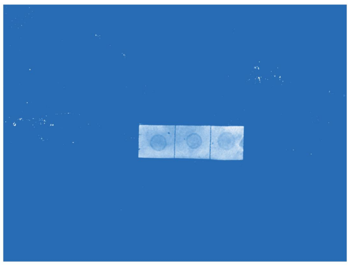

OVCAR3

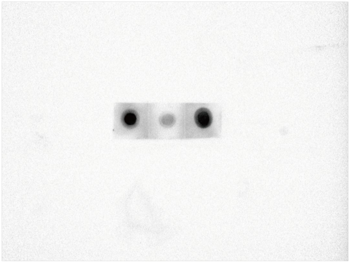

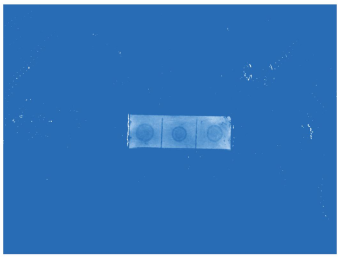

Figure3g

A2780

N-cadherin

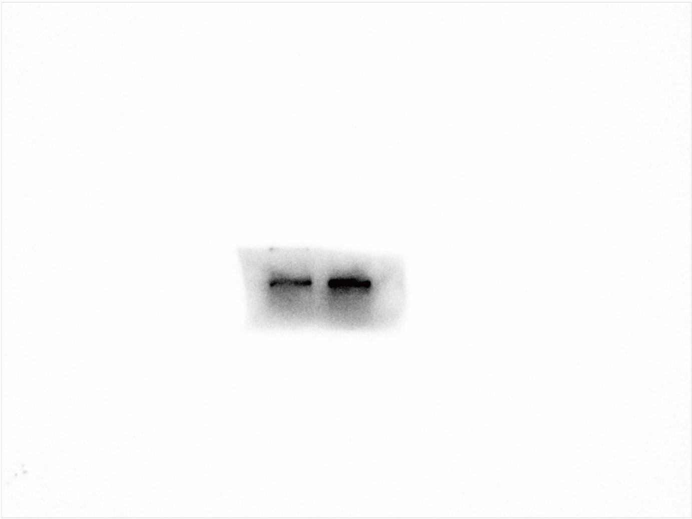

GAPDH

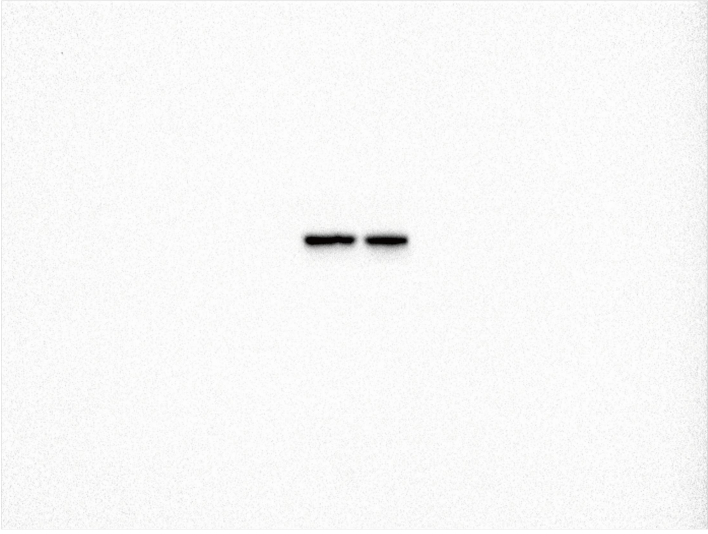

FTO

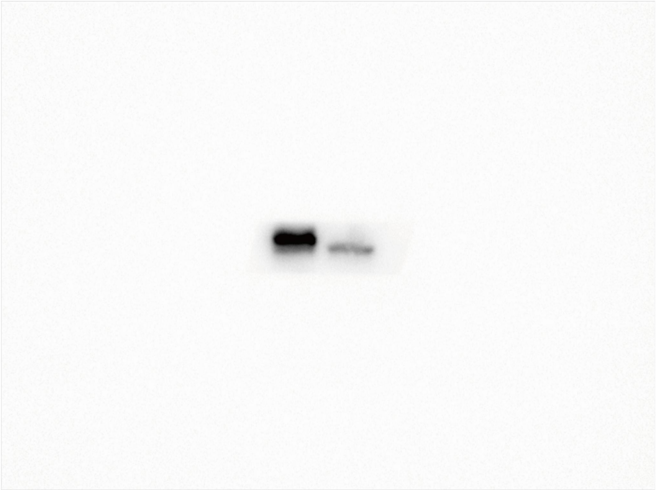

E-cadherin

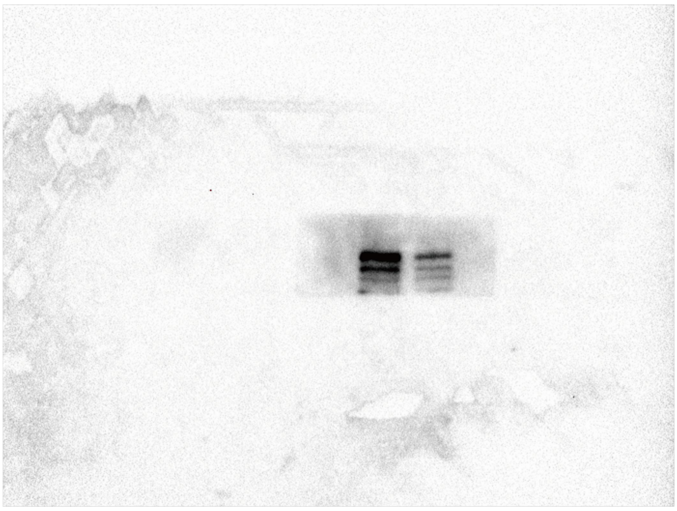

vimentin

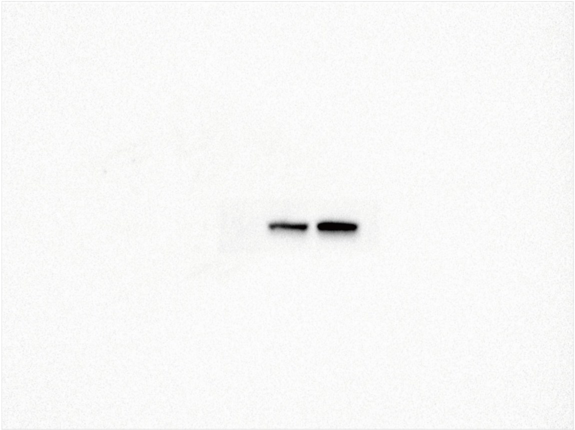

Figure3g
OVCAR3
N-cadherin

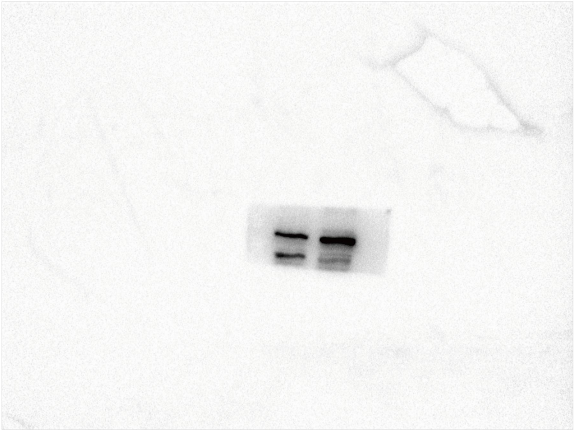

GAPDH

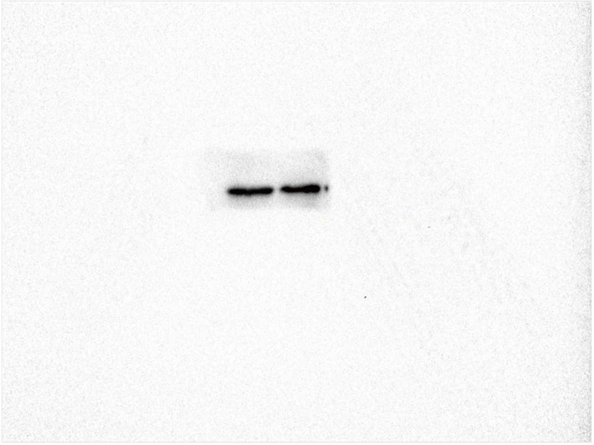

FTO

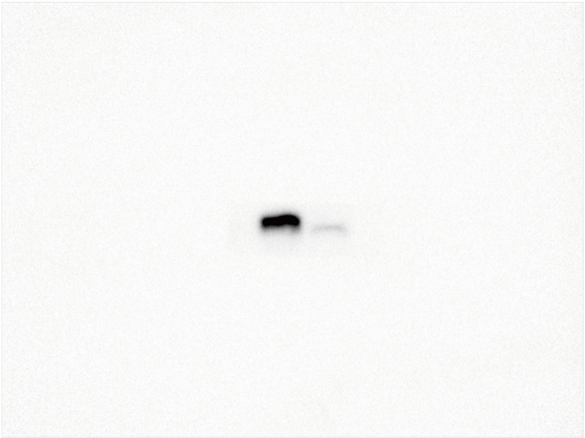

E-cadherin

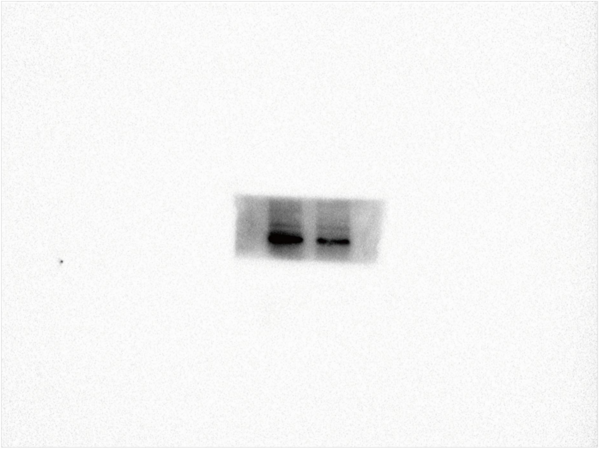

vimentin

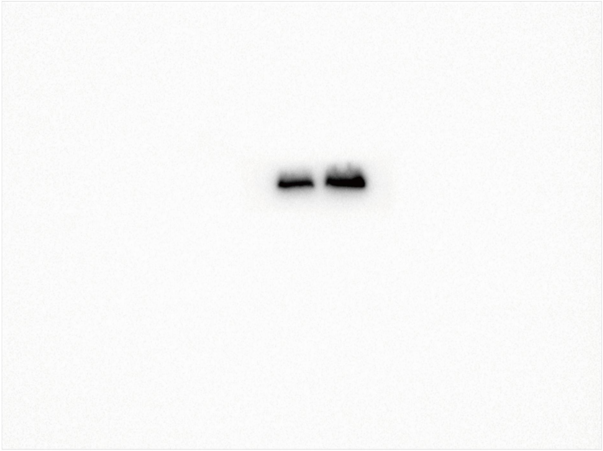

Figure4a

OVCAR3

ZEB2

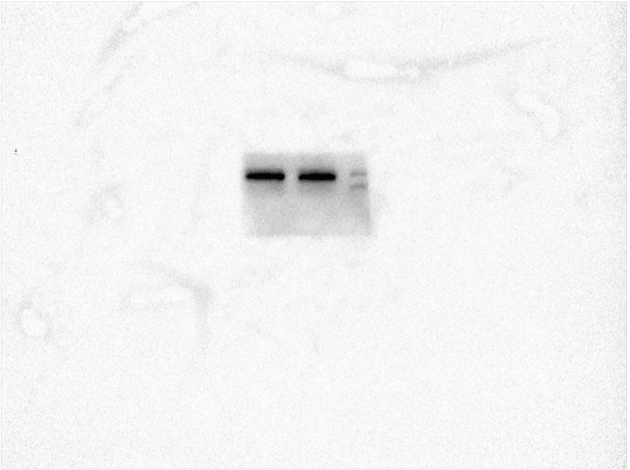

ZEB1

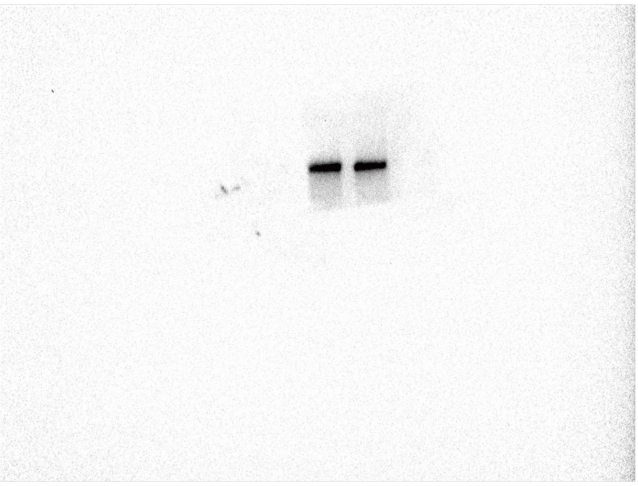

SNAIL

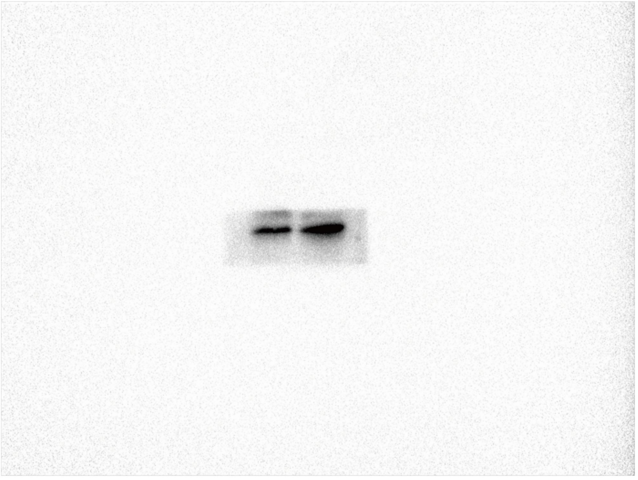

SLUG

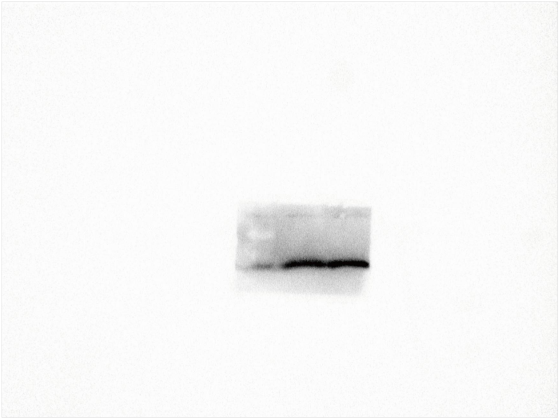

FTO

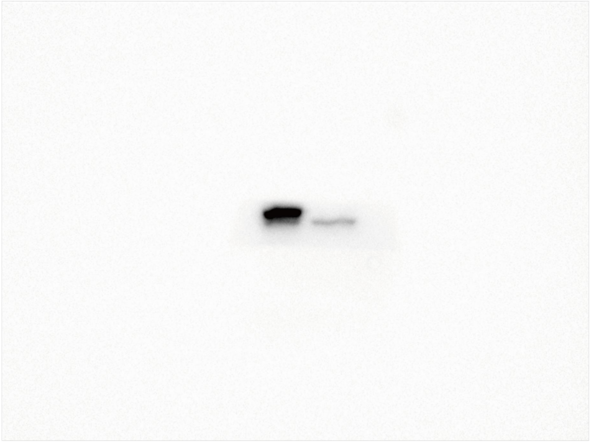

Beta-actin

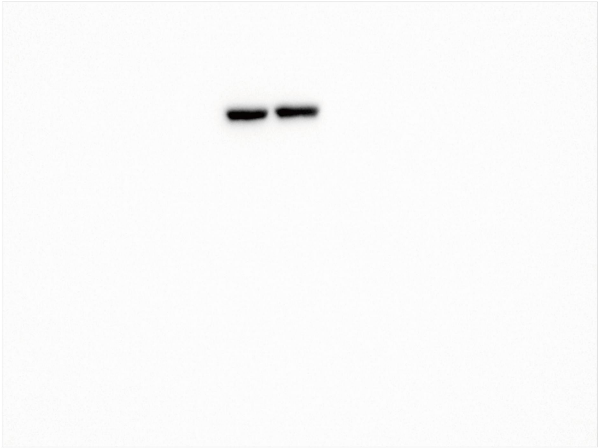

Figure4b

A2780

GAPDH

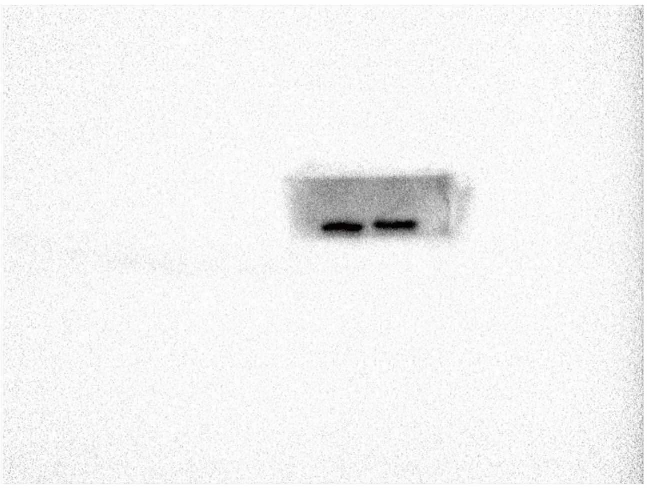

FTO

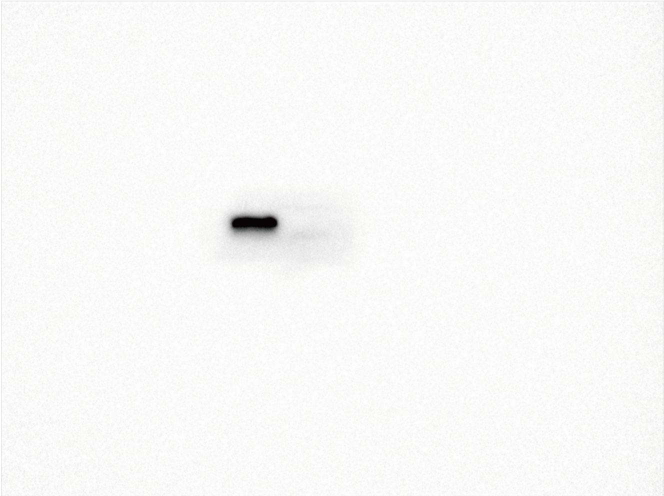

SNAIL

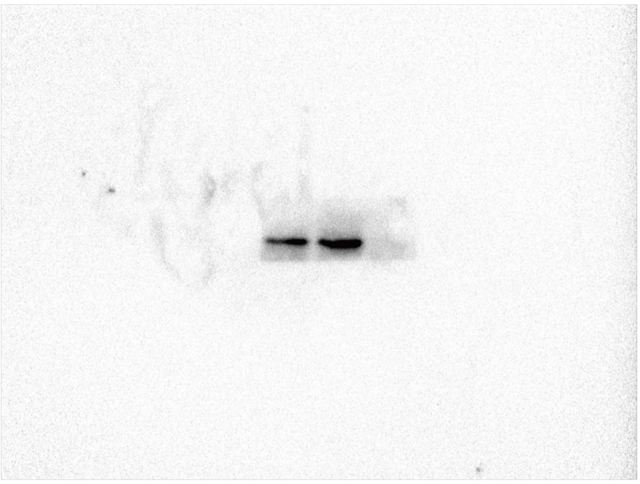

Figure6e

SNAIL

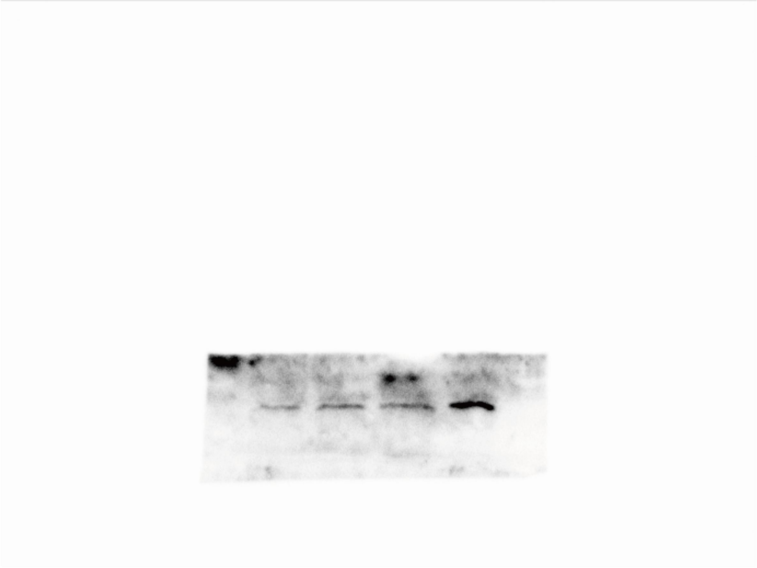

N-cadherin

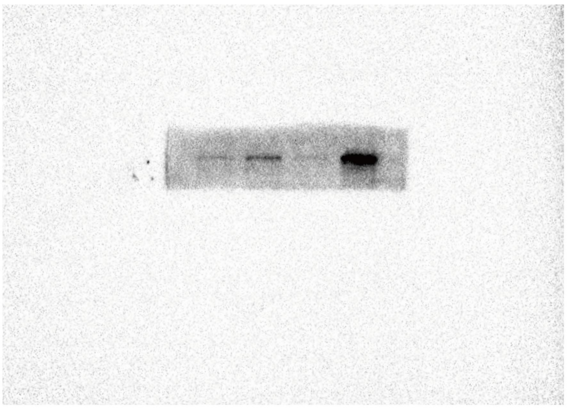

IGF2BP2

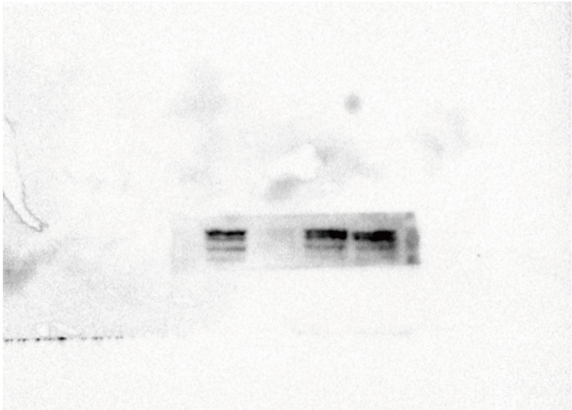

GAPDH

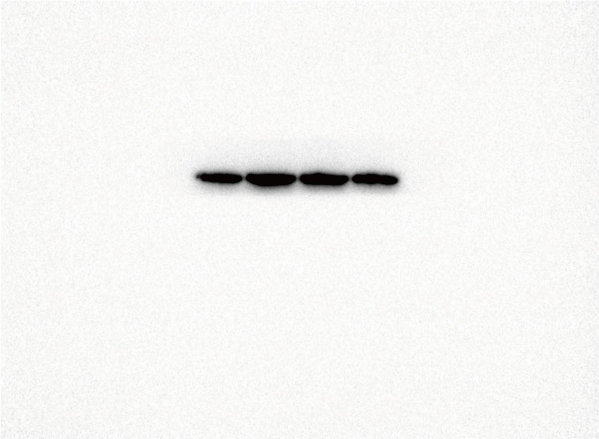

FTO

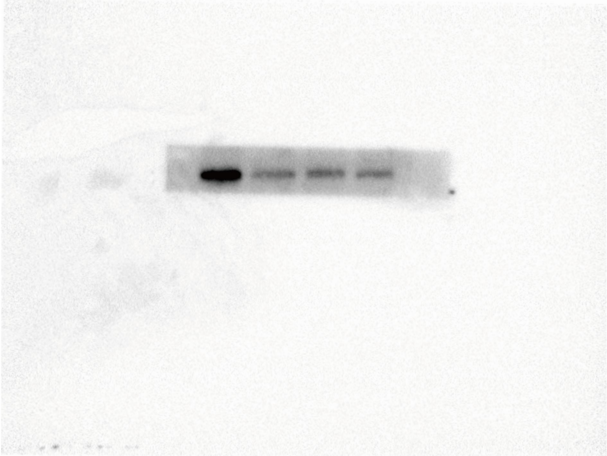

E-cadherin

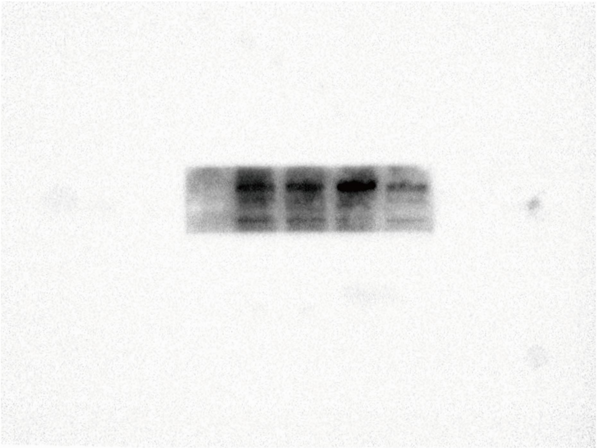

vimentin

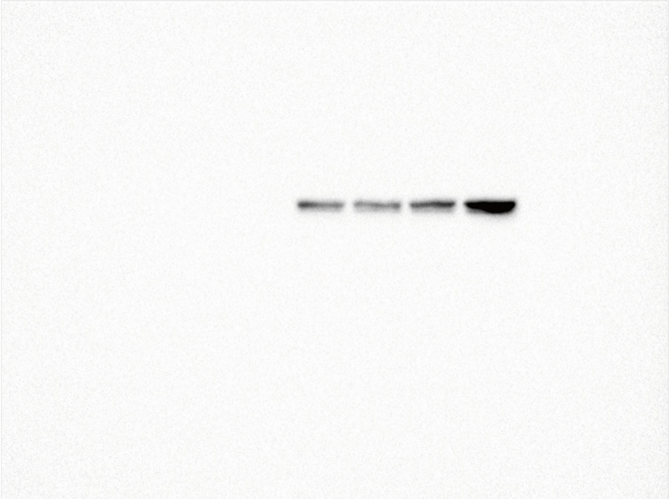

Figure7b

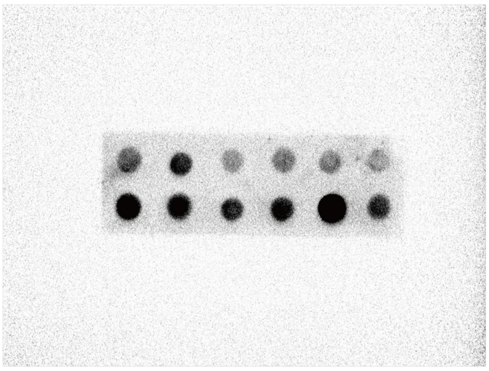

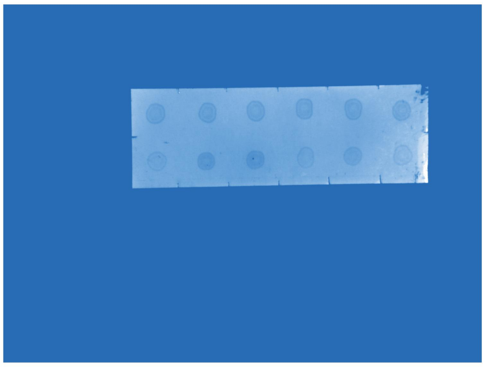

Figure7e

SNAIL

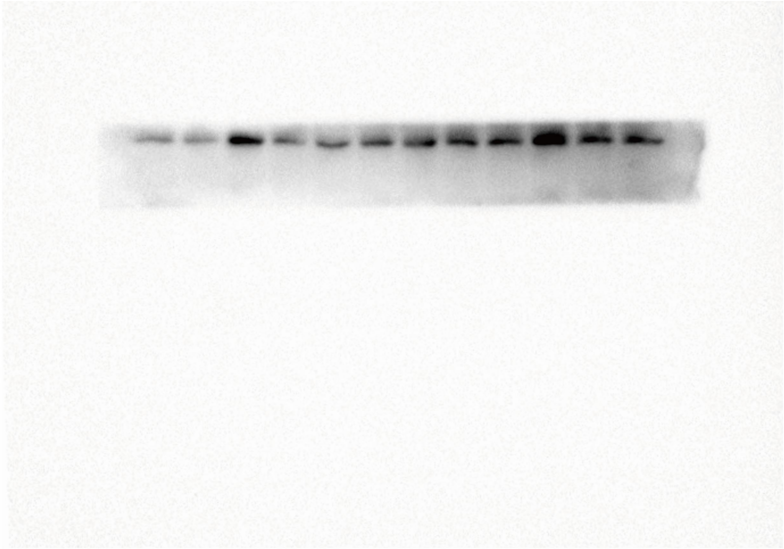

N-cadherin

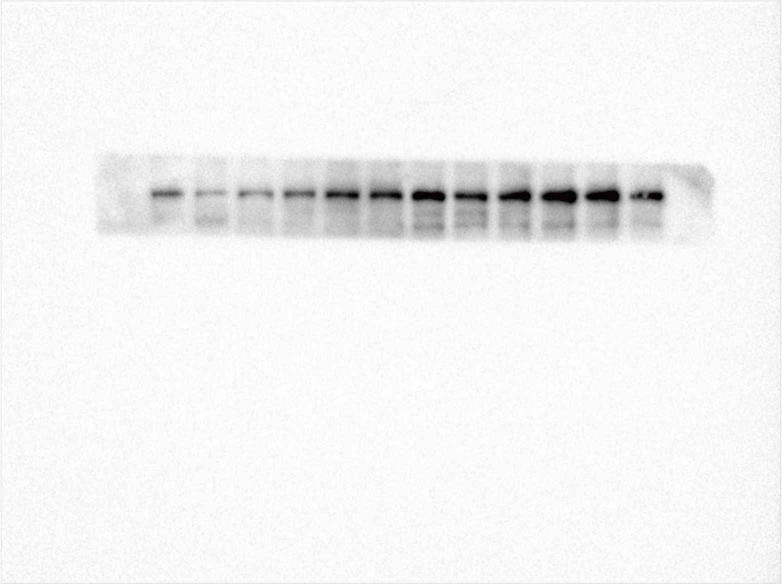

IGF2BP2

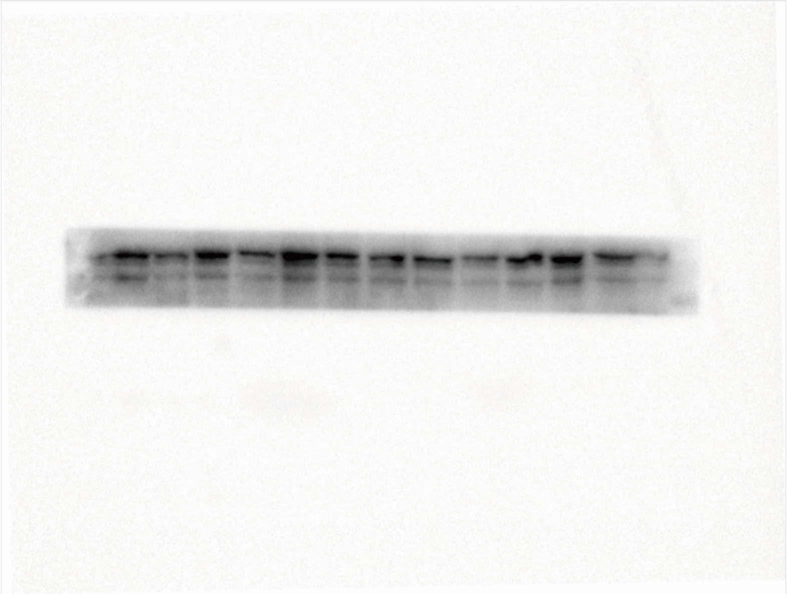

GAPDH

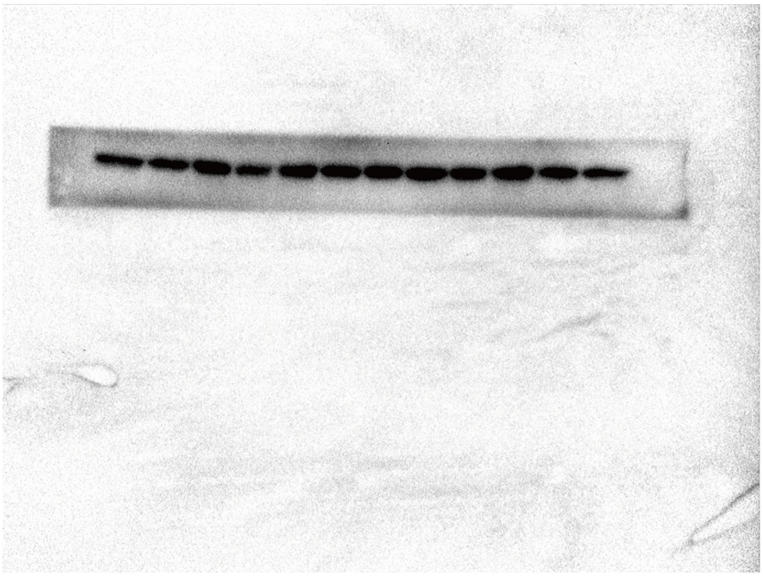

FTO

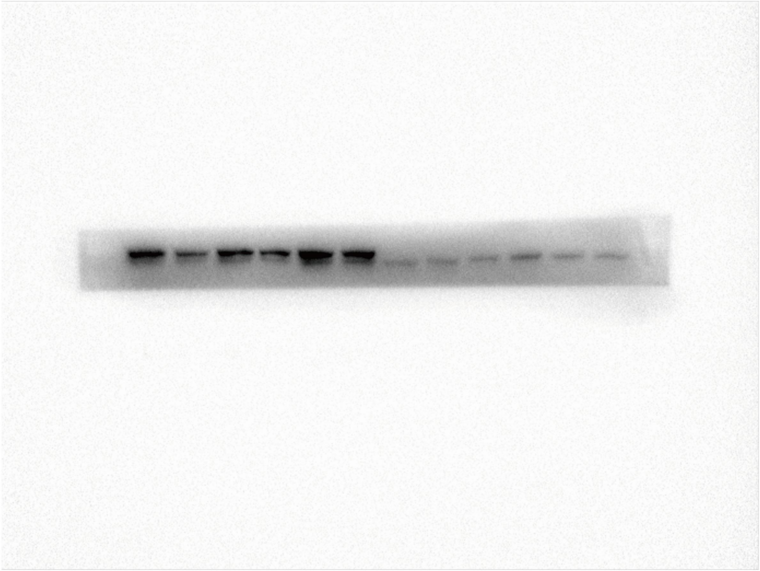

E-cadherin

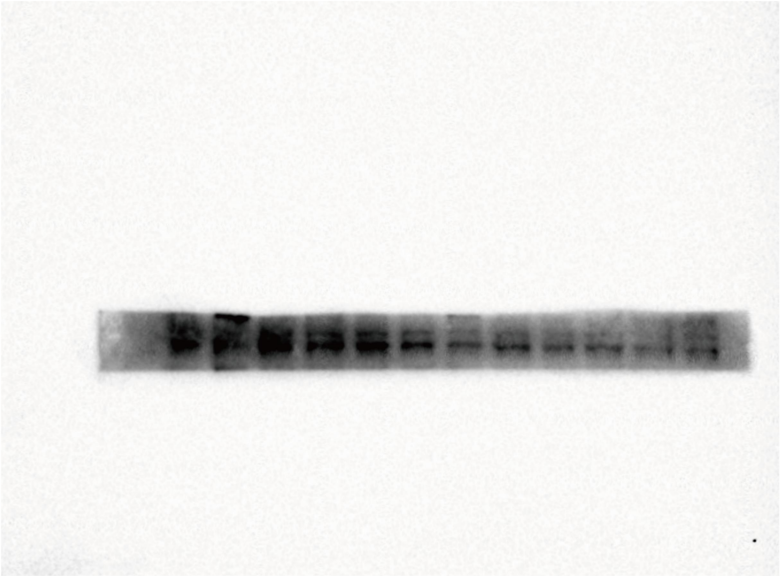

vimentin

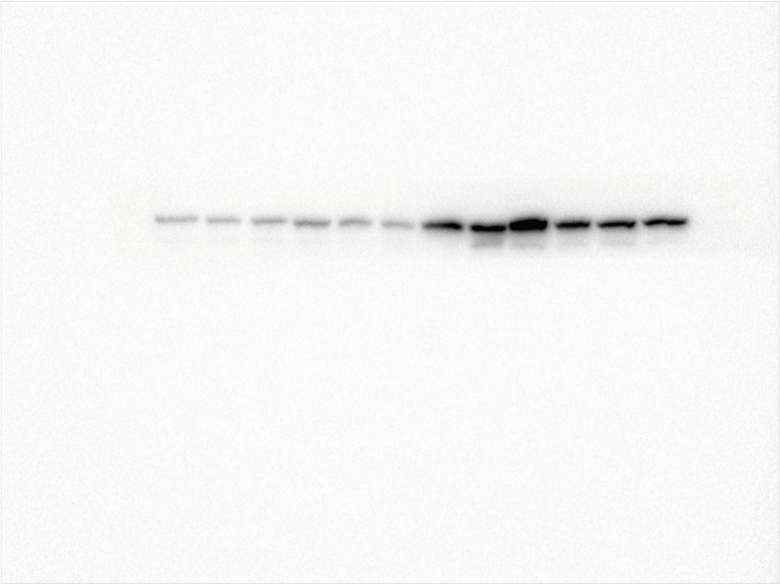

Supplement: Supplementary file 1 [file cancers-14-05218-s001.zip › supplementary file S1.pdf]
