# Supplementary material for: FTO Inhibits Epithelial Ovarian Cancer Progression by Destabilising SNAI1 mRNA through IGF2BP2
Source: Cancers (Basel). 2022 Oct 25;14(21):5218. doi: 10.3390/cancers14215218 (PMC9658695; doi:10.3390/cancers14215218)
Supplement: Supplementary file 1 [file cancers-14-05218-s001.zip › Table S1 Primer sequences.pdf]

	Forward (5' to 3')	Reverse (5' to 3')
GAPDH	CAGGAGGCATTGCTGATGAT	GAAGGCTGGGGCTCATTT
FTO	GTTCACAACCTCGGTTTAGTTC	CATCATCATTGTCCACATCGTC
SNAI1	TCTGAGCGGTGAGGGTTAGTGAG	CACGAAGGAAGAGAAATGGGTAGCC
METTL3	GTCAGGGCTGGGAGACTAGGATG	CAATGCTGCCTCTGGATTCCGTAG
METTL14	ACCAAAATCGCCTCCTCCCAAATC	AGCCACCTCTTTCTCCTCGGAAG
WTAP	AGGGCAACACAACCGAAGATGAC	ACCACTACCTCCTCTGCCAGTTC
ALKBH5	TCCTTTCCCTTCCCTTCTCCACTG	TGAAGCGGAGGAGGCACCAG
IGF2BP1	GATGAAGGCCATCGAAACTTTC	GGGGTGGAATATTTCGGATTTG
IGF2BP2	GATGAACAAGCTTTACATCGGG	GATTTTCCCATGCAATTCCACT
IGF2BP3	GAGGCGCTTTCAGGTAAAATAG	AATGAGGCGGGATATTTCGTAT
SNAI1-m6A#1	AGCAGGAAGGACCCCACA	CAGTGAGAAGGATGTGGG
SNAI1-m6A#2	GGAGGCGGTGGCAGACTA	GGGCATCTCAGACTCTAG
SNAI1-m6A#3	ACTCAGGGGACCCCACTC	GCCTGAGGGTTCCTTGTG
