# Supplementary material for: FTO Inhibits Epithelial Ovarian Cancer Progression by Destabilising SNAI1 mRNA through IGF2BP2
Source: Cancers (Basel). 2022 Oct 25;14(21):5218. doi: 10.3390/cancers14215218 (PMC9658695; doi:10.3390/cancers14215218)
Supplement: Supplementary file 1 [file cancers-14-05218-s001.zip › Table S2 transfection sequences.pdf]

	sense（5'-3'）	antisense（5'-3'）
si-FTO#1	GGCAAUCGAUACAGAAAGUTT	ACUUUCUGUAUCGAUUGCCTT
si-FTO#2	GUGGCAGUGUACAGUUAUATT	UAUAACUGUACACUGCCACTT
si-IGF2BP1	GCUCCCUAUAGCUCCUUUATT	UAAAGGAGCUAUAGGGAGCTT
si-IGF2BP2	CAGUUUGAGAACUACUCCUTT	AGGAGUAGUUCUCAAACUGTT
si-IGF2BP3	GCUGCUGAGAAGUCGAUUATT	UAAUCGACUUCUCAGCAGCTT
si-SNAI1	GCCUUCAACUGCAAAUACUTT	AGUAUUUGCAGUUGAAGGCTT
